# Towards a bioengineered airway: advances in tracheal tissue engineering and biofabrication

**DOI:** 10.3389/fcell.2026.1713743

**Published:** 2026-06-16

**Authors:** Palla Ranga Prasad, Praveen Kumar Sahni, Debadrita Mondal, Kirthanashri S. Vasanthan, S. Varadharajan, Ashwini Kumar, N. B. Shridhar, Naveena A. N. Kumar, Bharti Bisht, Kallyanashis Paul, Shayanti Mukherjee, Manash K. Paul

**Affiliations:** 1 Department of Radiation Biology and Toxicology, Manipal School of Life Sciences, Manipal Academy of Higher Education, Manipal, India; 2 Manipal Centre for Biotherapeutics Research, Manipal Academy of Higher Education, Manipal, India; 3 Manipal Institute of Technology, Manipal Academy of Higher Education, Manipal, India; 4 Department of Forensic Medicine, Kasturba Medical College, Manipal Academy of Higher Education, Manipal, India; 5 Department of Pharmacology and Toxicology, Obscure Disease Research Center, Veterinary College Campus, Shivamogga, India; 6 Department of Surgical Oncology, Kasturba Medical College, Manipal Academy of Higher Education, Manipal, India; 7 Department of Microbiology, Kasturba Medical College, Manipal Academy of Higher Education, Manipal, India; 8 The Ritchie Centre, Hudson Institute of Medical Research, Clayton, VIC, Australia; 9 Department of Obstetrics and Gynaecology, Monash University, Clayton, VIC, Australia; 10 Division of Pulmonary and Critical Care Medicine, David Geffen School of Medicine, University of California Los Angeles (UCLA), Los Angeles, CA, United States

**Keywords:** 3D bioprinting, bioink, reconstruction, regulatory framework, scaffold, tissue engineering, trachea

## Abstract

Tracheal damage arising from inflammation, trauma, congenital anomalies, or tumors can lead to life-threatening complications, yet current treatments, including surgical reconstruction, stenting, laser therapies, autografts, and allografts, remain inadequate, especially for long-segment defects. As a result, tracheal tissue engineering has emerged as a promising alternative, aiming to create functional biomimetic constructs that reduce dependence on complex surgeries, long-term stenting, and immunosuppression. Advances in additive manufacturing and 3D bioprinting have accelerated progress toward engineered tracheal substitutes; however, a fully functional, clinically viable 3D-bioprinted human tracheal graft has yet to be realized. This review assesses current bioengineering strategies, with a particular emphasis on the interplay between cell sources, scaffold materials, and fabrication methods, specifically focusing on 3D bioprinted tracheal constructs. Across existing studies, the most promising direction lies in multi-material, multicellular, spatially patterned bioprinting approaches that can better recapitulate the trachea’s complex biomechanics and heterogeneous tissue composition. Finally, we summarize regulatory considerations and outline key scientific and translational barriers, emphasizing that overcoming challenges in vascularization, innervation, and long-term functional integration will be essential to achieving a physiologically aligned, clinically deployable tracheal substitute.

## 1 Introduction

The trachea, or windpipe, is a flexible conduit connecting the nasal cavity to the lungs and facilitates the primary function of the lungs, i.e., inspiration and expiration (Furlow and Mathisen, 2018). Conditions like inflammation, tracheal trauma/injury, inborn abnormalities, and tracheal tumors (like lung squamous cell carcinoma, thyroid cancer, and adenoid cystic carcinoma) can lead to respiratory impairment, including airflow obstruction, coughing, difficulty breathing, stridor, and respiratory failure, and are associated with a high risk of mortality (Prange, 2019; Brand-Saberi and Schäfer, 2014). Commonly available treatment options for damaged or injured tracheal tissue are suboptimal laser treatment, stenting, grafting, and direct anastomosis. Laser therapy and tracheal stenting do not provide a long-term solution, but direct anastomosis for short tracheal defects and grafting for long tracheal defects may provide a permanent solution. Hence, a need for tracheal substitutes, which may be in the form of synthetic prosthesis, allografts/autografts, tracheal transplantation, and tracheal tissue bioengineering approaches. Tracheal grafting entails the transfer of tracheal tissue, or dermal grafts resected from the forearm/thigh/chest, and fortifying them with the patient’s rib, cartilage from the ribs, or parts of the removed trachea, which may be obtained from self, i.e., the patient’s tissue (autograft) or another source (allograft), to substitute or restore impaired sections of the trachea. Allografting is restricted due to the availability of donor tissues and the need to maintain a patient on an immunosuppressive drug regimen (Dhasmana et al., 2020). Among the several tracheal substitutes, tracheal autograft is currently being implemented to address long tracheal defects (Graziano et al., 1967). Segmental tracheal reconstruction involves resection and reconstruction via end-to-end anastomosis, which also faces substantial clinical challenges due to the lack of ideal tracheal replacement materials and the structural complexity of the trachea. Several approaches are used in clinical practice, like skin/fascia, polymer tube, mesh gauze, silicone tube, aortic auto/allograft, decellularized allograft, and reconstructed tissue composite, but limited options exist for long-segment tracheal anomalies (Greaney and Niklason, 2021).

Moreover, pediatric tracheal restoration must account for the patient’s growth and may need further operations or re-grafting for inert implants. Despite significant efforts, the limited results emphasize that restoring a fully operating trachea is complex and challenging and needs cutting-edge technology intervention. With the increasing footprint of respiratory disorders and their significant impact on the morbidity and mortality rates worldwide, tracheal defects and the need for novel tracheal substitutes are expanding, making this topic highly relevant. Nowadays, the emergence of tissue engineering approaches opens up promising potential to generate artificial tracheal grafts with airway-specific functions. The three central components of tissue engineering are cells, scaffolds, and growth factors, suitable for regenerating the desired tissue (Ott et al., 2011; Arnason, 2019). Among the different cell types, stem cells have been widely used in tissue regeneration because of their proliferative and self-renewal characteristics, and understanding stem cell biology is critical to developing tissue engineering trachea. Appropriate biomaterials must be chosen because the mechanical properties, biodegradation, bioactivity, biocompatibility, and architecture of the scaffolds also impact tracheal tissue regeneration. It is not only the scaffold material but also the selection of growth factors that regenerate particular tissue types, such as the trachea. Various growth factors like epithelial growth factor (EGF), platelet-derived growth factor (PDGF), basic fibroblast growth factor (bFGF), vascular endothelial growth factor (VEGF), granulocyte colony-stimulating factor (G-CSF), transforming growth factor (TGF), and insulin-like growth factor (IGF) have been used for tracheal tissue engineering (Fishman et al., 2014). Aside from the selection of cells, materials, and growth factors, fabrication techniques are also crucial for tracheal bioengineering.

Decellularization, electrospinning, and 3D bioprinting are the most commonly used techniques for tracheal tissue engineering. Currently, the majority of research work points to the advantages of 3D bioprinting over other traditional scaffold-fabricating techniques because it has a better ability to create intricate tissue structures through heterogeneous deposition of cells and biomaterials in a precise layer-by-layer manner. Bioink is a printable, cell-compatible material composed of biomaterials, growth factors, and living cells, often used in 3D bioprinting to fabricate biological tissues or organ-like constructs. It must provide structural support during printing while maintaining cell viability, promoting tissue development, and enabling functional maturation after printing (Gungor-Ozkerim et al., 2018). Currently, no 3D bioprinted clinically translatable functional tracheal biomimetic substitute exists as a long-term solution for tracheal defects. This lack of advancement may be ascribed to a multitude of issues, such as scientific failures, inflammation and tissue granulation at the transplantation site, unstable biomechanical properties of the degradable polymers, and incomplete neo-vascularization and neo-cartilage formation at the site of graft implantation. This review will focus on the different approaches to tracheal tissue engineering, with a specific emphasis on 3D bioprinting-based approaches for tracheal bioengineering, and aims to provide a comprehensive analysis of available data, focusing on existing challenges that need to be overcome to make a tissue-engineered product for patients with tracheal defects.

## 2 Anatomy and physiology of trachea

**FIGURE 1:**
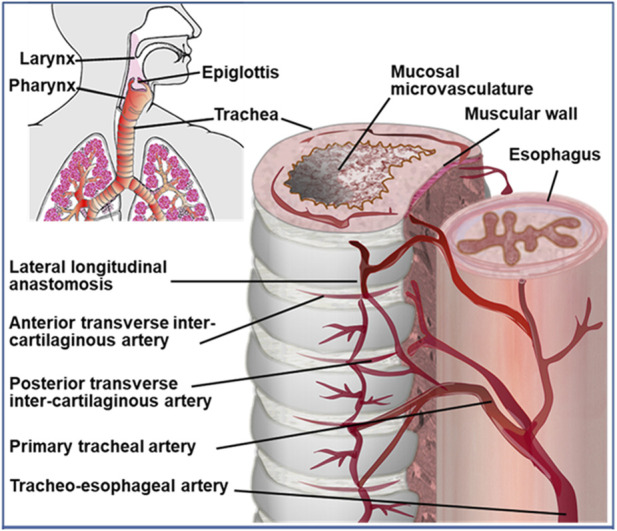
Anatomical features and vascular supply within the trachea. The tracheoesophageal arteries branch at the tracheoesophageal groove into esophageal and tracheal branches. The tracheal branches divide into superior and inferior segments, connecting with nearby arteries to ensure a steady blood supply to the tracheal rings. The esophageal branches also supply blood to the membranous part of the trachea (Remade the image inspired by (Furlow and Mathisen, 2018).

**FIGURE 2:**
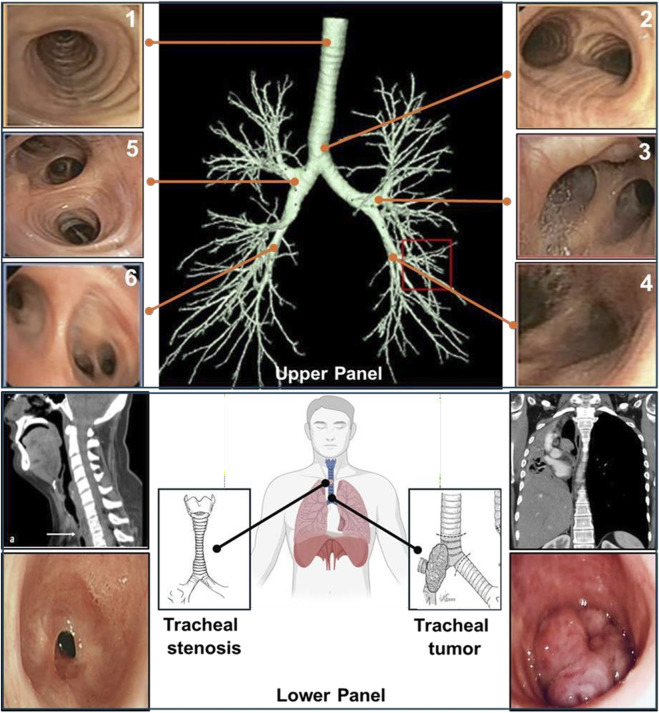
Upper Panel: Bronchoscopy images and process of airflow in the tracheobronchial tree. (1) trachea, (2) carina, right main bronchus, and left main bronchus, (3) right upper lobe bronchus (three apical segments), (4) right segment bronchus (six), (5) left upper lobe bronchus, (6) left segment bronchus (six). The tracheobronchial tree extends from the trachea to the terminal bronchioles. Air enters the carina, the right and left main bronchi from the trachea. This region is solely responsible for conducting air and does not participate in gas exchange. From the main bronchus, the air enters the upper lobe bronchi (right and left) and segment bronchi (right and left). It then exits the bronchus and enters the respiratory bronchioles through the terminal bronchioles. These respiratory bronchioles further divide into alveolar ducts, which are lined with alveoli. Lower Panel: CT scan, Bronchoscopy, and diagrammatic representations of tracheal stenosis and tracheal tumor. The trachea, or windpipe, is a pliable tube composed of cartilage that links the nasal passage to the lungs, facilitating the process of inhaling and exhaling in the human body. Tracheal diseases such as tracheal stenosis and tracheal tumors pose significant clinical challenges due to their impact on airway function and respiratory health (Patwa and Shah, 2015; Díaz-Jiménez and Rodríguez, 2023; Takata et al., 2022; Draeger et al., 2022; Jung Y.-R. et al., 2016; Grillo et al., 2002; Kumar et al., 2020).

The trachea is a hollow, semiflexible, tube-like structure ranging from 10 to 14 cm long and 1.5–2 cm wide (Brand-Saberi and Schäfer, 2014). Anatomically, the trachea/airway is a tube-like structure that begins under the larynx (voice box), extends behind the breastbone, and bifurcates into the “right and left main stem cell bronchi” (Mammana et al., 2024). The anatomy of the trachea is a typical D-shaped structure consisting of 16–20 incomplete C-shaped hyaline tracheal cartilaginous rings held together by fibro-elastic tissue (Figures 1, 2). The average height of each tracheal ring is ∼4 mm, whereas the outer diameter is ∼ 2–2.2 cm in men and 1.8–2 cm in women (Furlow and Mathisen, 2018; Minnich and Mathisen, 2007). The trachea has segmental vasculature, where arteries laterally enter the organ wall and connect with inferior and superior segmental arteries longitudinally. Each segmental artery splits into posterior and anterior branches, enveloping the trachea and connecting with the contralateral side of the segmental artery (Figure 1) (Furlow and Mathisen, 2018; Mammana et al., 2024). The tracheal wall has four layers: mucosa, submucosa, musculo-cartilaginous, and adventitia (Stick, 2012). The mucosal layer of the trachea is lined by pseudostratified columnar epithelium that contains basal stem cells, goblet, serous, club, and multiciliated cells, held by lamina propria, which contains tracheal or submucosal glands (SMG) (Figure 1) (Brand-Saberi and Schäfer, 2014). Some of the functions of the tracheal mucosa are mucus clearance, acting as a barrier, and initiating an immune response against respiratory pathogens (Rosso et al., 2016). The tracheal submucosa comprises stromal cells, fat cells, elastic fibers, loose connective tissue, lymphatic vessels, blood vessels, nerves, and many small mixed glands (seromucous glands) (Allen, 2003; Sommerhoff and Finkbeiner, 1990). The musculo-cartilaginous layer comprises tracheal muscle, fibroelastic tissue, and cartilaginous plates. The cartilaginous plates are composed of hyaline cartilage surrounded by perichondrium. Vascular channels are also consistently found in cartilaginous plates and can be identified by incision or trauma (Stick, 2012). The adventitia is the trachea’s outermost layer, consisting of connective tissue that combines with the musculocutaneous layer and surrounds the trachea. It also consists of loose areolar tissue that allows the free movement of the trachea.

The extracellular matrix (ECM) is the released product of native cells of the tracheal tissue, which makes it ideally suited to enable cell growth and homeostasis (Ravindra et al., 2021). Furthermore, the tracheal ECM contains elastin and collagen fibres, essential for ECM construction, remodelling, maintenance, and regenerative activities. Elastin and collagen are the main structural proteins of the ECM, thereby regulating the ECM matrix’s biological profile and the mechanical behaviour of the tracheal tissues (Trabelsi et al., 2010). In the trachea, elastic fibers are widely dispersed and well-organized, with distinct longitudinal bundles in the submucosa layer of the tracheal wall and widespread longitudinal fibres in the non-membranous areas located deep inside the cartilage rings (Kamel et al., 2009). In the case of collagen fibres, they are not oriented clearly. The thin tracheal layers near the lumen are oriented either longitudinally (along the airway axis) or circumferentially (along the cartilage). Meanwhile, they are oriented irregularly in the deepest layer and have a circumferential tendency (Teng et al., 2008). Normal tracheas broaden and extends with each breath. Wang et al. reported that alterations in the orientation and quantity of these fibres also have an impact on some biomechanical characteristics, namely tissue stiffness and expandability in the trachea (Wang et al., 2000).

Effective and safe tracheal reconstruction requires extensive knowledge of the tracheal vascular supply to minimize complications from tracheal ischemia (Furlow and Mathisen, 2018). Vascularization is one of the most critical factors in the trachea, as it provides nutrients and facilitates gaseous exchange in the human body (Salassa et al., 1977; Law et al., 2016). The trachea does not receive blood directly from the superior thyroid artery but through anastomosis with an inferior thyroid artery. Hence, the vascular supply of the trachea is divided into two regions: upper cervical and lower thoracic trachea (Figure 1). From inferior thyroid arteries, the third tracheoesophageal branch delivers the blood supply to the upper cervical trachea through the left and right thyrocervical trunks that split from the subclavian arteries.

Meanwhile, the middle cervical trachea receives it from the second branch, and the lower cervical trachea gets it from the first branch (Salassa et al., 1977). However, the branch configuration may vary significantly across individuals. The thoracic trachea receives blood from the bronchial arteries, which originate directly from the aorta. The superior, inferior, and middle bronchial arteries commonly deliver blood to the trachea (Furlow and Mathisen, 2018). The trachea also receives blood from small branches originating from the internal mammary, innominate, and subclavian artery. When the tracheoesophageal branches reach their groove, they divide into primary esophageal and primary tracheal branches. The tracheal arteries enter the trachea through its lateral wall and divide into inferiorly and superiorly throughout the length of numerous tracheal rings (Figure 1). The trachea has an extensive submucosal plexus nourished by inter-cartilaginous arteries penetrating the soft tissue between the tracheal rings. As arteries reach the midline, they go deeper and end in the submucosal capillary plexuses. These plexuses deliver the blood supply to the tracheal cartilages, whereas secondary branches originate from primary esophageal arteries and vascularize the membranous trachea (Drevet et al., 2016).

The trachea, bronchi, and bronchioles together form the human tracheobronchial tree, a complex and branched network system that carries air from the outside to the lungs for gaseous exchange (Florens et al., 2011). This system consists of 23 generations or branches, beginning from the trachea and ending in the terminal bronchioles, while each generation divides one airway into two smaller daughter airways (Figure 2) (Patwa and Shah, 2015). In a healthy human, the average length and diameter of the tracheobronchial tree are L_o_ = 12 cm and D_o_ = 1.8 cm, respectively (Florens et al., 2011). The tracheobronchial airway system comprises around 1% of the total lung capacity, and the rest of the lungs consist of large blood vessels and parenchyma, including interalveolar septa, alveolar airspaces, and bronchioles (Hyde et al., 2009). During respiration, cartilage is essential for avoiding airway collapse. As we go down to the tracheobronchial tree, the amount of cartilage decreases, whereas the smooth muscle amount increases. The smooth muscle also helps to regulate the airflow through airway contraction and dilation. Due to a lack of cartilage, elastic fibers, and smooth muscles maintain the integrity of the bronchioles wall (Patwa and Shah, 2015). The bronchial arteries from the descending aorta supply blood to the tracheobronchial tree from the carina to the respiratory bronchioles (Widdicombe, 2010). Hence, overcoming these challenges might provide a long-term solution for tracheal bioengineering.

## 3 Tracheal diseases and their surgical procedures

The architecture of the trachea plays an essential role in maintaining the airway system by providing structural support and allowing the clearance of mucus and debris (Minnich and Mathisen, 2007). Tracheal disorders and diseases, including tracheal stenosis, tracheomalacia, and tracheal tumors are among the most common conditions that significantly impair airway functions (Mathisen, 1998). These diseases and disorders can arise from various factors, including inflammation, congenital defects, infections, external trauma, injury during surgical procedures, and other causes (Muthialu et al., 2020). Tracheal stenosis is a condition where the narrowing or constriction of the trachea or windpipe occurs, preventing air from fully entering the lungs (Figure 2) (Kumar and Salib, 2006). The primary causes of tracheal stenosis include tracheostomy or prolonged intubation, where a tube is used to facilitate breathing via a mechanical ventilator. Other contributing factors include external throat injury, infections, autoimmune disorders, the growth of malignant or benign tumors exerting pressure on the trachea, and adverse effects from radiation therapy during cancer treatment (Puchalski and Musani, 2013). Tracheal stenosis can be diagnosed through various tests, including pulmonary function tests, bronchoscopy, chest and neck computed tomography (CT) scans, magnetic resonance imaging (MRI), and X-rays of the trachea and chest (Smith and Cotton, 2018). Various surgical procedures are employed to treat tracheal stenosis, depending on its cause, location, and the degree of narrowing. Bronchoscopic dilation involves widening the trachea using a balloon, electrocautery, or a tracheal dilator. This technique provides immediate symptom relief and also helps to determine the severity of the stenosis (Nikolovski et al., 2019). Laser bronchoscopy resects scar tissue with lasers for short-term relief (Shah and Chalam, 2009). The tracheobronchial airway stent, or T-tube, is a non-invasive treatment that uses a bronchoscope to place a stent, keeping the airway open (Wood et al., 2003). Tracheal resection and reconstruction involve removing the scarred and narrowed tracheal segment and rejoining the upper and lower regions. This procedure offers long-term outcomes and is the preferred surgical option for certain types of stenosis and tumors (Donahue et al., 1997).

Tracheomalacia is a rare disorder characterized by the weakening and collapse of the tracheal cartilage walls, resulting in airway obstruction (McNamara and Crabbe, 2004). Common causes include inflammation of the tracheal cartilage, chronic infections, injury during surgical procedures, prolonged external ventilation, and gastroesophageal reflux disease (Hysinger, 2018). Diagnosis of tracheomalacia is typically performed using bronchoscopy, pulmonary function tests, MRI, chest and neck CT scans, and chest X-rays (McNamara and Crabbe, 2004). Various surgical procedures are available for treating tracheomalacia. Tracheostomy is a surgical process that supports the collapsed trachea by tightening the surrounding tissue, repairing the floppy airway, and preventing collapse (McNamara and Crabbe, 2004). Tracheoplasty or tracheal stenting involves reinforcing the tracheal walls with autologous tissue or synthetic biomaterials to enhance structural support to the trachea and prevent airway collapse (Wright, 2015). For severe cases of tracheomalacia, tracheal resection and reconstruction are performed. This procedure involves removing the narrowed or damaged tracheal segments and reconnecting the healthy ends, restoring the trachea’s structural integrity and function (El-Sayed et al., 2009).

Airway or tracheal tumors are sporadic and can cause constriction of the trachea, preventing air from entering the lungs. These tumors are classified into benign and malignant forms and can significantly affect airway functions (Grillo, 2003). Papillomas, hemangiomas, granular cell tumors, and benign fibrous tumors are examples of benign tumors (Handa et al., 2016). In contrast, squamous cell carcinoma, adenoid cystic carcinoma, malignant epithelial tumors, and non-small cell lung cancer are examples of malignant tumors (Grillo, 2003). Smoking is the primary risk factor for the development of tracheal tumors (Harris et al., 2018). In addition to smoking, tracheal tumors can also arise from various other risk factors, including chronic inflammation and infections, physical trauma or injury, long-term exposure to certain chemicals, and tumor recurrence following radiotherapy. Diagnostic techniques include bronchoscopy, CT scan, chest radiographs, MRI, and PET scans to detect tracheal tumors (Karlan et al., 2016). Various surgical procedures are employed in the treatment of tracheal tumors. Tracheobronchial stenting is a minimally invasive procedure that alleviates airway obstruction caused by tracheal or airway tumors. It involves placing a stent, typically made of silicone and other materials, into the trachea to maintain airway openness and provide immediate relief. This intervention enhances the quality of life for patients with tracheal tumors and can serve as a bridge to more advanced treatments (Pang et al., 2006). A tracheostomy is a vital surgical procedure involving an incision through the neck into the trachea, followed by the insertion of a tube to create an air passage. This tube allows air to bypass the obstructed segment caused by a tumor, ensuring proper oxygenation and ventilation. This approach quickly and effectively secures the airway, providing a temporary solution before the definitive tumor excision (Grillo, 1978). Nowadays, tracheal resection and reconstruction is a complex but highly effective surgical procedure for the treatment of tracheal tumors. These procedures aimed to remove a tumor-affected segment of the trachea and reconnect the healthy ends to restore normal airway functions. The end-to-end anastomosis must be tension-free to facilitate wound healing and avoid complications (Kumar et al., 2020).

Surgical procedures for tracheal disorders or diseases often do not provide a permanent solution due to several limitations. While they may provide immediate relief and temporary restoration of airway function, they frequently come with significant risks such as infection, granulation tissue formation, stent migration, and recurrence of the original condition. Additionally, the mechanical nature of these interventions can lead to further complications and may not address the underlying pathology, resulting in repeated surgeries and ongoing medical management. Moreover, only a few skilled surgeons worldwide are capable of performing these intricate and time-consuming operations. The tracheobronchial system also shows various anatomical variations, such as tracheal bronchus and accessory cardiac bronchus, with a prevalence of 4% (Abakay et al., 2011). It is currently unknown how common these variations are because they do not cause any symptoms. Therefore, it is crucial to identify these variations before performing specific post-operative procedures, such as endotracheal intubation, bronchoscopy, and lung isolation techniques (Drevet et al., 2016). Hence, there is a need for an alternative strategy to regenerate the trachea.

## 4 Tissue engineering for tracheal replacement

Tissue engineering has arisen as a possible solution for the regeneration of various types of tissues. Recently, it has gained more attention in respiratory medicine, specifically for tracheal tissue reconstruction and replacement, offering the potential for long-term and even permanent solutions (Ramos and Moroni, 2020). These approaches involve using bioengineered scaffolds, stem cells, and growth factors to create a biologically compatible trachea that can integrate with the patient’s native tissue. By promoting the regeneration of functional tracheal tissue, these techniques aim to restore the natural structure and function of the airway, reducing the risk of complications associated with synthetic materials and mechanical devices. Moreover, regenerative approaches can address the root cause of the tracheal disorder by healing and rebuilding the affected area, potentially eliminating the need for further surgical interventions. Moreover, for pediatric cases, it can bring significant relief. This innovative field is poised to revolutionize the treatment of tracheal diseases, providing more effective and sustainable solutions for patients.

Tracheal tissue engineering is the recreation of an artificial tissue-like tracheal substitute for tracheal regeneration using tissue engineering approaches (Dhasmana et al., 2020). Although, tissue-engineered approaches have the potential to provide an optimal tracheal substitute. However, limited clinical data is available to establish the effectiveness of tissue engineering techniques for tracheal replacement. Despite several attempts being made to implant an artificial tissue-engineered trachea in humans as a therapy, the outcomes are inconsistent and unfavorable (Stocco et al., 2023). The main goal of tissue engineering strategies is to develop a non-immunogenic substitute by using patients' own stem cells to derive a fully functional respiratory mucociliary epithelium with ideal mechanical characteristics for the regeneration of tracheal tissue (Grillo, 2002). Various research studies have started focusing on tracheal regeneration (Fuchs et al., 2002; Kojima et al., 2003; Zang et al., 2012; Zani et al., 2008) and have started identifying and addressing the challenges associated with tissue-engineered tracheal regeneration. Some of the current applications and promising approaches might provide a foundation for developing the gold-standard tissue-engineered tracheal construct, which will be discussed in the next section.

## 5 Approaches to tracheal tissue engineering

**FIGURE 3:**
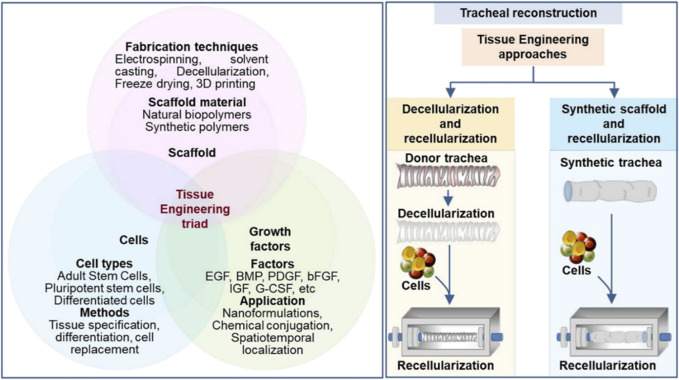
Fundamental components and strategies in tracheal tissue engineering. The left panel highlights the tissue engineering triad: scaffolds, cells, and growth factors. Scaffolds are made from natural or synthetic polymers, provide structural support are fabricated using methods like electrospinning, decellularization, and 3D printing. Cells, including adult stem cells, pluripotent stem cells, and differentiated cells, enable tissue regeneration through processes like specification, differentiation, and replacement. Growth factors such as EGF, BMP, and PDGF regulate cellular behavior and are delivered using nano-formulations and spatiotemporal control. The right panel depicts two tracheal reconstruction approaches: one involves decellularizing a donor trachea to retain the ECM, followed by recellularization with a combination of the above-mentioned cells. The other utilizes a synthetic scaffold, also seeded with cells, to replicate native tissue function. Both methods aim to restore airway integrity and function [Figure inspired by Dhasmana et al. (2020)].

The tracheal tissue comprises several cells, such as epithelial, endothelial, chondrocytes, and smooth muscle cells (Baiguera et al., 2010a). As mentioned below in Figure 3, we illustrate the diverse approaches employed in tracheal tissue engineering. The figure highlights the different types of scaffolds, signaling molecules, and stem cell sources used to support tracheal regeneration. It also depicts the range of fabrication and recellularization techniques applied to reconstruct functional tracheal segments. Together, these elements provide a comprehensive visual overview of current strategies in tracheal bioengineering.

### 5.1 Cells for tracheal bioengineering

**FIGURE 4:**
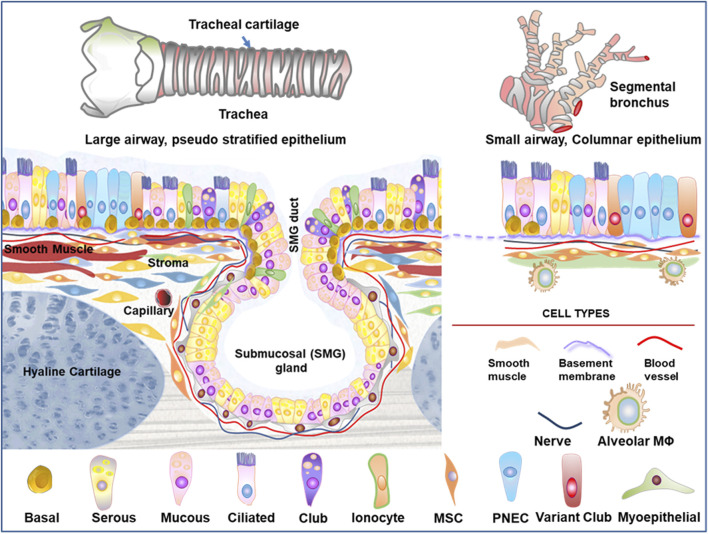
Schematic of tracheal structure highlighting its layered architecture, cell types, and functional microenvironment components. The trachea consists of four layers: mucosa, submucosa, cartilaginous layer, and adventitia. The mucosal layer includes ciliated epithelial cells, basal cells, serous cells, mucous cells, and Clara (club) cells, which aid in airway protection and mucociliary clearance. Beneath it, the submucosa contains seromucous gland cells, fibroblasts, mesenchymal stem cells (MSCs), and smooth muscle cells, providing structural support and regeneration. The cartilaginous layer has chondrocytes in cartilage rings, ensuring airway rigidity. The adventitia, encasing the layers, includes fibroblasts, blood vessels, adipose tissue, and nerves, facilitating vascularization and flexibility (Dayanand et al., 2022; Dhasmana et al., 2020).

Reconstructing the trachea requires recapitulating a diverse set of cell types, each with a unique and essential function in maintaining airway structure and homeostasis (Figure 4) (Yeou and Shin, 2025). Airway basal stem cells (ABSCs) are the primary adult stem cells in the airway epithelium, marked by Krt5+, Tp63+, PDPN+, NGFR+, and Krt14+ (Dayanand et al., 2022). ABSCs can lead to the formation of ciliated (Foxj1+, acetylated β-tubulin+), club (Scgb1a1+, previously known as Clara), goblet-1 (MUC5AC+, MUC5B+), goblet-2 (ZG16B+), tuft (TRPM5+, DCLK1+), ionocyte (CFTR+, FOXI1+), and pulmonary neuroendocrine (Ascl1+, CHGA+) cells (Wu et al., 2022). Within them, “hillock” cells express Krt13+ and aid in forming squamous epithelium (Montoro et al., 2018). Epithelial cells regulate airway physiology and act as a physical barrier. Tracheal respiratory epithelium and nasal tissue are the primary sources of epithelial cells for tracheal regeneration; however, induced pluripotent stem cells (iPSC)-based approaches can also be considered (Kojima et al., 2003). Epithelial cells seeded on a scaffold show cell viability and enhanced tracheal tissue regeneration *in vivo* (Bae et al., 2018; Go et al., 2010). The airway-resident stromal cells mainly comprise of pericytes (PDGFRβ+, NG2+), smooth muscle cells (α-SMA+, MYH11+), fibroblasts (FAP+, Vimentin+, CD90^+^), endothelial cells (CD31^+^, CD34^+^), and mesenchymal cells (CD90^+^, CD73^+^, CD105+). Tracheal stromal cells, including fibroblasts and MSCs, are essential for tracheal regeneration, driving ECM deposition, remodeling, and biomechanical integrity crucial for airway patency and resilience (Nahumi et al., 2022). Fibroblasts in the lamina propria and posterior membranous region produce collagen, elastin, and proteoglycans, which contribute to flexibility and strength, while also releasing paracrine cues that facilitate epithelial maturation and vascular growth (PMID: 23671810). MSCs derived from bone marrow, adipose tissue, or iPSC sources improve graft performance by modulating inflammation, promoting angiogenesis via VEGF and bFGF secretion, and differentiating into cartilage-like or myofibroblastic cells that support vascularization, epithelial regeneration, and long-term graft survival (Liang et al., 2025). Airway hyaline cartilage primarily maintains airway patency, comprises chondroblasts and chondrocytes (ACAN+, COL2A1+, and Sox9+) (housed in spaces called lacunae) and the extracellular matrix (ECM) (Barreiro Carpio et al., 2021). Chondrocytes are primarily present in the cartilaginous tissues, such as the nasal, auditory, costal septum, and bone (Walles et al., 2004). Tracheal chondrocytes play a crucial role in tracheal reconstruction by producing hyaline cartilage C-rings that provide essential mechanical support to the airway (Shin et al., 2014). These rings prevent collapse under negative pressure while allowing flexibility in the posterior membranous area (Han et al., 2022). In normal physiology, they generate an extracellular matrix rich in type II collagen and proteoglycans, which provides the airway with its anisotropic stiffness, radial rigidity, and longitudinal flexibility, necessary to maintain the lumen open during breathing (Gomes et al., 2025). In tracheal bioengineering, co-culture strategies systematically integrate multiple cell types to replicate the structure and function of native tissue, addressing the limitations of monocultures by promoting coordinated epithelial-mesenchymal interactions essential for mucociliary differentiation and biomechanical functionality integrity (Yeou and Shin, 2025). Various co-culture formats are used, including direct and indirect (transwell) systems, spheroid-based co-cultures, cell sheet engineering, heterogeneous 3D-bioprinted constructs, and more complex tri- or multi-cellular combinations (Yeou and Shin, 2025). Direct contact co-culture approaches facilitate close cell–cell communication through gap junctions and promote ECM remodeling, essential for epithelial polarization and chondrogenesis. Micromass co-cultures of MSCs-derived chondrocytes generate cartilage-like nodules with increased collagen II/X and SOX9 expression (Janardhanan et al., 2012), while pellet cultures combining nasal chondrocytes and fibroblasts enhance GAG production via paracrine FGF signaling. Similarly, epithelial–mesenchymal co-cultures using airway epithelial cells and tracheal fibroblasts promote basement membrane maturation (laminin and collagen IV gradients) and robust ciliation within 21 days, recapitulating native tracheal signaling essential for mucociliary function (Ishikawa et al., 2017). Doolin et al. co-cultured human epithelial cells with articular cartilage on a fibrin-glue-based scaffold, resulting in a fully functional tracheal construct. This construct maintained viability for 2 weeks *in vitro*, forming an intact epithelial monolayer that directly adhered to cartilage sheets without delamination, thereby preserving airway lumen integrity. Implanted heterotopically in nude mice for 4 weeks, the grafts showed preserved cellular architecture, ECM deposition, and absence of necrosis, establishing proof-of-concept for adhesive-mediated tissue engineering of tubular airway structures without sutures or synthetic scaffolds (Doolin et al., 2002). Indirect co-culture systems rely on paracrine communication, which occurs without direct cell contact, making them well-suited for examining temporal differentiation. Transwell co-cultures of MSCs with airway epithelial cells exhibit VEGF-driven vascularization and enhanced epithelial proliferation (Luengen et al., 2022). In contrast, when combined with small airway epithelial cells, bone marrow-derived MSCs increase ALDH3A1 expression through conditioned media, thereby enhancing xenobiotic defense (de Hilster et al., 2024). Similarly, scaffold-based (PCL/alginate) indirect co-cultures of chondrocytes and endothelial cells stimulate angiogenesis through the secretion of MMP13 and PTHrP, thereby helping to alleviate hypoxia in thicker tissue constructs (Kundu et al., 2015). Kojima et al. developed a functional tracheal equivalent by co-culturing sheep nasal epithelial cells with chondrocytes and seeding them onto polyglycolic acid scaffolds. The scaffolds were molded into cylindrical rings using silicone templates and cultured in chondrogenic media for 4 weeks, resulting in the formation of mature cartilage. Epithelial cells were then seeded onto the luminal surface and matured under air–liquid interface conditions for 2 weeks, producing a pseudostratified, mucin-secreting epithelium. Following *in vitro* analysis, the constructs were implanted subcutaneously into athymic nude mice for 6 weeks, showing ECM remodeling, maintained luminal patency without collapse, and host integration with no inflammatory response. This work demonstrates the feasibility of multi-material, multi-cellular tissue-engineered constructs for airway reconstruction (Kojima et al., 2003). Spheroid-based co-cultures combine multiple cell types, such as epithelial, MSC, and endothelial cells, in the ratios of 4:1:1 using methods like magnetic levitation or hanging drops, allowing them to fuse into trachea-like rings with emerging self-vascularization and perfusable networks (Machino et al., 2019). Using Kenzan robotic bioprinting, spheroids of size 200–500 μm can be precisely positioned into lattice structures that mature into perfusable tracheal constructs with approximately 80% fusion efficiency and active ECM remodelling, including collagen I and IV deposition. Co-spheroid systems additionally promote early mucociliary differentiation, evidenced by increased FOXJ1+ precursor cell formation (Moldovan et al., 2017). Temperature-responsive polymer dishes allow intact epithelial or chondrocyte sheets to be harvested without enzymatic dissociation, preserving cell–cell junctions and native ECM for multilayer stacking. When chondrocyte sheets are wrapped around epithelial sheets, they form tubular constructs with over 90% cell viability and mechanical anisotropy that resembles the native trachea (Kanzaki et al., 2006). Similarly, hTMSC sheets combined with nasal epithelial cells generate a well-differentiated, ciliated pseudostratified epithelium after 4 weeks at the air–liquid interface (Park et al., 2018). Multi-material bioprinting enables precise spatial organization of different cell types, such as incorporating PCL frameworks with nasal chondrocyte–based cartilage rings, tracheal epithelial layers from hNTSCs, and HUVEC-lined vascular channels. Dual-extrusion techniques produce bellows-style grafts with distinct zonal architecture, achieving cartilage stiffness and epithelial barrier function (Yu et al., 2025). Hybrid FDM–DLP printing of PCL–SilMA constructs, seeded with fibroblasts, supports robust pre-vascularization following maturation in the omentum (Lee et al., 2024).

Endothelial-inclusive co-culture systems help overcome the major challenge of hypoxia in engineered tracheal constructs. HUVEC–MSC–fibroblast spheroids (1:3:1) generate hierarchical vascular networks, while GelMA constructs printed with chondrocytes and endothelial cells successfully anastomose with host vessels in rabbit tracheal defects, maintaining patency for up to 8 weeks (Shafiee et al., 2021). Additionally, airway smooth muscle–epithelial co-cultures demonstrate functional phenotype switching, where epithelial secretomes drive ASM cells from a proliferative to a contractile state (Tugade and Ramis, 2025). In Table 1, we have summarized the co-culture strategies in tracheal bioengineering, showing cell source combinations, their biological outcomes, advantages, and limitations. It compares how each approach aids epithelial regeneration, cartilage formation, vascularization, and graft function, highlighting their potential and challenges for clinical tracheal constructs.

**TABLE 1:** Summary of various co-culture strategies used in tracheal bioengineering, highlighting their outcomes, advantages, and limitations.

Co-culture strategy	Cell source combinations	Key outcomes	Advantages	Limitations	References
Direct contact	MSCs + chondrocytes, epithelial cells + fibroblasts, epithelial cells + chondrocytes	Increased SOX9 expression, collagen II/X, and GAG production, preserving basement membrane and airway lumen integrity	Promote ECM remodelling, epithelial polarization, and chondrogenesis	Overgrowth and less nutrient diffusion	Janardhanan et al. (2012), Ishikawa et al. (2017), Doolin et al. (2002)
Indirect (transwell)	MSCs + epithelial cells, chondrocytes + endothelial cells, epithelial cells + chondrocytes	Vascularization, enhanced epithelial proliferation, cartilage, and pseudostratified, mucin-secreting epithelium formation	Mimics *in vivo* communication, facilitates studies on paracrine signalling, and supports tissue engineering efforts	No spatial organization and signalling dilution	Kojima et al. (2003), Luengen et al. (2022), de Hilster et al. (2024), Kundu et al. (2015)
Spheroid-based	Epithelial cells + MSCs + endothelial cells	Active ECM remodelling, collagen 1 and IV deposition, promotes early mucociliary differentiation	Self-vascularization and perfusable networks	Variability in spheroid sizes	Machino et al. (2019), Moldovan et al. (2017)
Cell sheet engineering	hTMSC + epithelial cells, chondrocytes + epithelial cells	Well-differentiated, ciliated pseudostratified epithelium, 90% cell viability, and mechanical anisotropy similar to native trachea	Preserving cell-cell junctions and native ECM for multilayer stacking	Thickness limit of the cell sheet and manual stacking scalability	Kanzaki et al. (2006), Park et al. (2018)
3D bioprinted heterogeneous	Chondrocytes + hNTSC + HUVEC	Distinct zonal architecture, supports pre-vascularization, achieving cartilage stiffness, and epithelial barrier function	Enable the precise spatial organization of multiple cell types	Decreased cell viability and poor bioink shear-thinning	Yu et al. (2025), Lee et al. (2024)
Tri/multi-cultures (vascular)	HUVECs + MSCs + fibroblasts, smooth muscle cells + epithelial cells	Proliferation of epithelial cells and maintains airway patency	Overcome hypoxia in engineered tracheal constructs	Allogenic, immunogenicity, and hypoxia core	Shafiee et al. (2021), Tugade and Ramis (2025)

**FIGURE 5:**
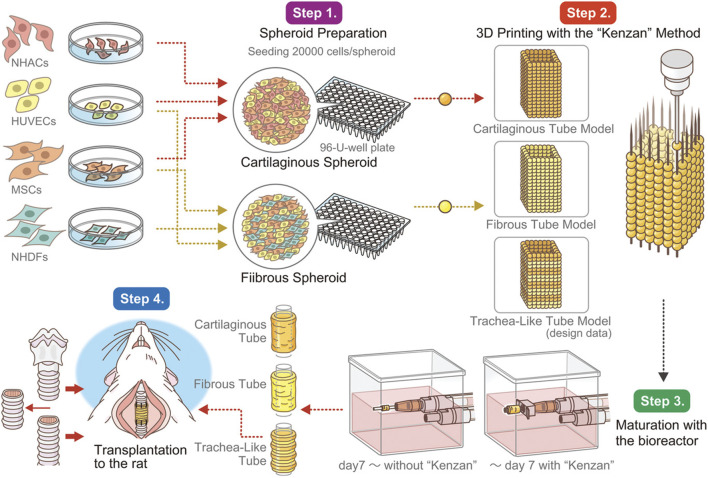
Substitution of rat tracheas with scaffold-free, multi-layered frameworks of human cells using a Bio-3D printing system. An illustrative diagram depicting the process of generating the tubes. Step 1: Preparation of spheroids using four different cell types. Step 2: Bio-3D printing was conducted using the “Kenzan” technology, which included the creation of three different kinds of tubes. Step 3: Facilitate maturation by ensuring the appropriate flow of the medium. Step 4: Transplantation into the rat. Figure used with permission (Machino et al., 2019).

In the past, chondrocytes and specific polymers were used to create tissue-engineered tracheal bioprosthetics for implantation. The current methods for tracheal tissue regeneration primarily use autologous chondrocytes and mesenchymal stem cells (MSCs)-derived chondrocytes. Machino et al. co-cultured MSCs isolated from bone marrow with umbilical endothelial cells, fibroblasts, and cartilage to create cell-aggregated spheroids. They printed these spheroids into 3D tubular structures resembling native tracheal tissue. Figure 5 illustrates the complete workflow for generating scaffold-free, multilayered human cell–based tracheal grafts using Bio-3D printing with Kenzan technology and their subsequent transplantation into a rat model (Machino et al., 2019). According to a recent study by Knaneh-Monem et al. and colleagues, when smooth muscle cells are co-cultured with human fetal organoids, a fully functional neo-tissue is formed that exhibits epithelial proliferation and differentiation-associated ciliary movement (Knaneh-Monem et al., 2019). With the rapid advancement of our understanding and multifaceted roles of airway stem cells in epithelial regeneration, the functional heterogeneity of stromal fibroblasts (Aros et al., 2020), MSCs in ECM remodelling and neovascular support, and hyaline chondrocytes in maintaining mechanical stability. It has become imperative that, successful tracheal bioengineering needs multi-lineage, and strategies to spatially organize cells. Another factor is the use of coordinated paracrine signaling and zonal control for accurately mimicking the native airway. Collectively, these scientific and technological advancements are poised to significantly accelerate clinical translation of tracheal tissue grafts (Al Shetawi et al., 2021).

### 5.2 Biomaterials for tracheal bioengineering

The scaffold is a crucial component in the success of tissue engineering techniques. For tracheal tissue regeneration, an ideal scaffold should possess specific characteristics such as having a porosity of approximately 90% and a pore size ranging from 5 to 100 μm for better cell survival, proliferation, and vascularization. It should be designed to imitate the shape of a defect in the trachea or native tracheal tissue. A suitable scaffold should also have good flexibility (10.6 ± 1.8 MPa) and mechanical strength (212 ± 18 N) to prevent collapse and ensure proper functioning. It should also be biodegradable to support gradual host remodeling. The scaffold must be biocompatible and non-toxic, supporting cell adhesion and proliferation without inducing cytotoxic effects. Equally important is its non-immunogenic nature, as it should not trigger adverse host immune responses such as inflammation, fibrosis, antibody formation, or T-cell activation that could compromise graft survival (Chan and Leong, 2008). Together, these properties are essential to minimize rejection, promote effective tissue integration and neo-vascularization, facilitate regeneration of functional mucociliary epithelium, ensure mechanical stability and long-term airway patency, and ultimately achieve successful clinical reconstruction (Greaney et al., 2023; Liu et al., 2024). There are three types of biomaterials utilized for the regeneration of tracheal tissue such as natural biomaterials, which come from native biological tissue materials, synthetic biomaterials, which are made through synthetic means, and hybrid biomaterials where the combination of cells and polymers (natural or synthetic) are used. For tracheal tissue engineering, scaffolds can be made from a variety of polymeric materials, including decellularized matrix, collagen, fibrin, gelatin, alginate, silk fibroin, Pluronic F127, methacrylate, poly (ethylene glycol) (PEG), polycaprolactone (PCL), poly-lactic acid (PLA), polyglycolic acid (PGA), and poly-lactic-glycolic acid (PLGA). These materials can be utilized individually or in combination with others (Baiguera et al., 2010a; Hong et al., 2018). Hydrogel is another approach used for transplanting a combination of cells and polymers (Lee and Mooney, 2001; El-Sherbiny and Yacoub, 2013). Recently, scaffold-free constructs have gained attention in tracheal bioengineering, offering potential advantages in mimicking native tissue architecture without the interference of synthetic polymers. These constructs are self-assembled aggregates of cell structures formed through techniques like cell sheet engineering, magnetic levitation, and spheroid aggregation, where living cells, such as chondrocytes, epithelial cells, and MSCs, produce their own ECM without the use of exogenous biomaterials, enabling higher cell density, natural remodeling, and reduced foreign body responses (Taniguchi et al., 2018a; Wang L. et al., 2025; Yang et al., 2022). In Table 2, we have provided a comprehensive summary of the natural and synthetic biomaterials used in tracheal substitute engineering, outlining their unique properties, the fabrication techniques employed, and the outcomes reported in preclinical or experimental studies. Additionally, it clearly emphasizes the advantages and limitations of each biomaterial, facilitating an effective comparative assessment within the context of tracheal tissue engineering. Below is an overview of the various biomaterials employed in tracheal bioengineering.

**TABLE 2:** Comprehensive list of natural and synthetic biomaterials with unique properties, fabrication techniques, and outcomes for tracheal substitute engineering.

Biomaterials	Properties	Method	Pros	Cons	References
Decellularized Cadaveric Trachea	Ability to retain the ECM	Decellularization	Functional and proper tracheal graft without immune rejection	Restriction in vascular supply	Nahumi et al. (2022)
Decellularized human cadaveric trachea	Indirect revascularization	Decellularization	Fully vascularized and regenerated tissue	Applied to selected individuals with long-segment tracheal stenosis	Yeou and Shin (2025)
Collagen	Good biodegradable properties	Polymerization and stratification	Columnar ciliated epithelium formation and complete tracheal mucosa tissue regeneration	Further experimentations are required for clinical applications	Hutmacher (2001)
Collagen/polypropylene	Bipotential nature	Freeze-drying	Tracheal epithelium regeneration	Slow absorption of the transplanted collagen sponge	Wu et al. (2022)
Collagen/polypropylene	Good biomechanical and biodegradable properties	Freeze-drying, polymerization	Tracheal epithelial regeneration	Requirement of specified fibroblasts	Montoro et al. (2018)
Collagen and gelatin	Highly biocompatible and biodegradable	Freeze drying	Restore the tracheal functions	Lack of mechanical properties	Yamamoto et al. (2003)
Gelatin	Good biodegradable	Freeze drying	Epithelial tissue regeneration and chondrogenesis formation	Only applied to minor wound injuries	Özpolat et al. (2013)
Silk and titanium mesh	Good biodegradability, biocompatibility, and elasticity resistance	Electrospinning and thermostatic electric air-blowing drying	Formation of the tracheal mucosal layer with ciliated growth	Infiltration of plasma cells and a few fibroblasts at the site of incomplete silk fibroin absorption	Liang et al. (2025)
Silk fibroin and collagen	High tensile strength, mechanical, and differentiation properties	Electrospinning, polymerization, and vitrification	Formation of mature epithelial and tracheal tissue	Low-ciliated and goblet cell populations compared to native trachea physiological conditions	Barreiro Carpio et al. (2021)
Hylaograft C	Good biocompatible properties	3D bioprinting	Slow degradation of neocartilage	Graft failure	Subia and Kundu, (2010)
Fibrin and titanium mesh	High mechanical and biodegradable properties	Polymerization	Tracheal mucosal regeneration	Low fibrosis	Han et al. (2022)
PCL and sodium alginate	High biocompatible, biodegradable, and mechanical properties	3D bioprinting	Vascularization and cartilage formation	Graft failure	Bae et al. (2018)
PCL, gelatin, and collagen	Good biocompatible and biodegradable properties	Freeze-drying	Regeneration of tissue and mucosal lining	Lack of mechanical strength and graft rejection	Lin et al. (2008) Lin et al. (2009)
PCL and Pluronic F127	Enhanced mechanical and biodegradable properties	Immersion precipitation method	Promote mucous regeneration	Constriction at the anastomosis site and short lifespan of the graft	Janardhanan et al. (2012)
PGA and alginate	High porosity and degradation rate	Cross-linking	Mature tracheal cartilage formation	High mortality rate	Kundu et al. (2015)
PLGA and collagen	Highly bioabsorbable, biodegradable, and biomechanical	Freeze-drying and cross-linking	Better mechanical strength	Poor tracheal cartilage formation	Kanzaki et al. (2006)
PLGA	Enhanced biocompatible, biochemical, and mechanical properties	Solvent-casting method	Tracheal tissue regeneration	High degradation and deformation	Kanzaki et al. (2006)
PLGA, PLCL Collagen and silk	Enhanced mechanical, biocompatible, and biodegradability	Electrospinning	Significant cell proliferation, infiltration, and degradation	Further studies are required on the structural and functional simulation of tri-layer grafts	Yu et al. (2025)
PP and Dacron	Highly biocompatible and biophysical	Polymerization	Fully functional epithelial lining	No mucosal lining	Lee et al. (2024)
PP and silicone tube	Good mechanical properties	Polymerization	Neo-cartilage regeneration and vascularization	Minimal morbidity is achieved	Shafiee et al. (2021)
PP and collagen	Good mechanical properties	Gelation, vitrification, cross-linking	Regeneration of columnar ciliated epithelium	Studies on larger animal models with tracheal defects are required	Wu et al. (2022)
PU	Good biocompatible and biochemical properties	3D printing	Promote re-epithelization and connective tissue ingrowth with microvasculature	Lack of tracheal biomechanical functions	Knaneh-Monem et al. (2019)
PU and PET	Enhanced mechanical stiffness, degradability, and porosity	Electrospinning	Epithelial migration, neo-cartilage formation, and wound healing	Delayed stenosis	Aros et al. (2020)
PU, PET, and PC	Good biomechanical, porosity, and degradation properties	Electrospinning and 3D printing	Tracheal tissue regeneration is similar to native tissue	Studies on larger animal models are required	Wei et al. (2024)
PDMS	Topography and stiffness	Polymerization	Better cell viability and differentiation marker expression	Less cell viability	Al Shetawi et al. (2021)
HDP and Pluronic F127 hydrogel	Good mechanical, bioabsorbable, and biodegradable properties	Polymerization and crosslinking	New cartilage formation	Inflammation and incomplete mucosal regeneration	Hong et al. (2018)
PCL and decellularized ECM hydrogel	Good mechanical, biodegradable, and bioresorbable properties	3D printing and decellularization	Successful healing and complete re-epithelialization	Mild granulation at the anastomosis site	Lee and Mooney (2001)
PCL and PEG hydrogel	Good biodegradation and compatibility	Polymerization and freeze-drying	Retention of chondrogenic ability and neo-tracheal cartilage formation	Insufficient mechanical properties	El-Sherbiny and Yacoub (2013)
Sil-MA hydrogel	Good mechanical, biocompatibility, rheological, and biodegradability	Digital light processing 3D printing	Formation of cartilage-like tissue with epithelium	Limited printable biomaterials, limited range of light sensitivity	Taniguchi et al. (2018a)
Sil-MA hydrogel sheets	Enhanced structural integrity, mechanical, biodegradability, and biocompatibility	4D-bioprinting	Cartilage and epithelium formation	Cell culture medium time or type influences the shape morphing of cell-laden hydrogels	Poon (2018)

#### 5.2.1 Natural scaffold

Natural polymers are non-toxic, biodegradable substances similar to natural materials and possess inherent properties of natural restructuring, biomimicry, and bioactivity.

##### 5.2.1.1 Decellularized scaffold

Decellularized matrix-based scaffolds are intriguing bio-scaffolds that provide specific benefits, particularly for tracheal regeneration. A viable tracheal transplant can be generated by eliminating donor cells from the donated tracheal tissue (decellularization), resulting in an extracellular matrix (ECM) scaffold. Subsequently, the scaffold may be populated with the recipient’s cells (recellularization) to alleviate the likelihood of immunological rejection and enhance integration with the recipient tissue. In a study conducted on pigs, Go et al. successfully regenerated a decellularized cadaveric trachea by recellularizing it with tracheal epithelial stem and differentiated cells. The regenerated trachea was transplanted into the affected area without immune rejection, which maintained mucociliary clearance, structural integrity, and vascular ingrowth without rejection, ischemia, or tracheomalacia for up to 60 days (Go et al., 2010). In another preliminary study, Delaere et al. implanted decellularized-recellularized human cadaveric tracheal scaffolds that were pre-treated with stem cells and implanted into the forearm of the recipient. After 4 months, the scaffold had transformed into fully vascularized and regenerated tissue, which could be used to treat tracheal defects (Delaere et al., 2010). This strategy yields variable effects and requires numerous stages to achieve optimal patient outcomes. While it may provide functional tracheal replacements, further research and clinical trials are necessary to improve the procedures, results, and accessibility of this treatment.

##### 5.2.1.2 Collagen

Collagen is a protein-based polymer that plays a crucial role in the tracheal extracellular matrix (ECM) structure. Collagen may be combined with other ECM proteins and components, such as elastin, chitosan, hyaluronic acid, and chondroitin sulfate, to generate scaffolds with improved biological and mechanical features. Nomoto et al. conducted a study using a ring-shaped scaffold made of spongy collagen coated with collagen gel. They seeded the scaffold with tracheal epithelial cells to help tissue regeneration and accelerate wound healing. The researchers implanted the tracheal epithelial cells seeded collagen-based scaffold on a tracheal defect (measuring 1.5:3 dimensions) in rats. After 2 weeks, they observed the formation of columnar ciliated epithelium and complete tracheal mucosa tissue regeneration, offering a feasible acellular approach for mucosal defects suitable for partial tracheal reconstruction (Tada et al., 2012). In a different study, mice were implanted with a freeze-dried collagenase polypropylene scaffold. This resulted in the complete regeneration of epithelial tissues, mucosal lining, and ciliated cells, which act as a bipotential scaffold, offering a cell-free strategy for tracheal mucosal defects and bioengineered airway reconstruction (Tada et al., 2008). Nomoto et al., in a study, developed a bioengineered collagen-based spongy scaffold, which was implanted along with fibroblast and tracheal epithelial cells to regenerate rapid re-epithelialization in rats with tracheal defects via paracrine signals, improving bioengineered airway graft performance for stenosis repair without immunosuppression. The regenerated cells were analyzed histologically after 14 days of implantation (Nomoto et al., 2008). Omori et al. and his team reported the first successful clinical trial of a tissue-engineered tracheal transplant in a 78-year-old patient with thyroid cancer. The team created a ring-shaped scaffold using spongy collagen coated with collagen gel to mimic native tracheal tissue and replace the three damaged tracheal rings (right half) after scaffold implantation. Two months after the surgery, epithelial lining coverage was established and lasted for 2 years without complications. However, tissue regeneration with mucosal lining showed complete tissue epithelization within 2 months without granulation, stenosis, and infection (Omori et al., 2016). In another investigation, Komepel et al. used collagen-fibrin hydrogel combined with platelet-derived growth factor (PDGF) to heal tracheal defects (Delaere et al., 2010). As a result, the wound was covered with ciliated epithelium, but there was also high inflammation at the wound site (Koempel et al., 1998). Several researchers have designed gelatin and collagen-based scaffolds to regenerate tracheal tissue. These natural biopolymers are the primary organic constituents of tracheal tissue. Gelatin is essentially the denatured version of collagen protein. A study conducted by Okamoto et al. and Yamamoto et al. involved implanting freeze-dried collagen and gelatin-based scaffolds that were incorporated with growth factors into canine tracheal gaps. The findings showed that the grafts were able to restore the tracheal functions, but they lacked mechanical strength (Okamoto et al., 2003; Yamamoto et al., 2003).

##### 5.2.1.3 Silk

Silk is another natural biomaterial used to fabricate a scaffold for tracheal tissue engineering because of its good mechanical strength and biocompatibility. Ni et al. experimented on male white New Zealand rabbits to reconstruct tracheal defects. They used a scaffold made of silk fibroin coated with titanium mesh seeded with fibroblasts and implanted into the rabbits. After 3 months, radiologic and histopathological studies showed the presence of a tracheal mucosal layer with cilial growth, maintained patency, and mild inflammation at the implantation site (Ni et al., 2008). Varma et al. conducted a thorough comparative analysis of various biomaterials to create an appropriate bioengineered tracheal epithelial graft in another study. They found that the combination of collagen vitrigel membrane and silk fibroin shows better mechanical strength and cellular differentiation, forming mature epithelia and tracheal tissue (Varma et al., 2018).

##### 5.2.1.4 Gelatin

Gelatin, which is obtained from collagen, is a versatile substance used for scaffolding. It has been studied for its potential to regenerate the trachea owing to its biocompatibility, biodegradability, and capacity to activate cell attachment and growth. Ozpolat et al. conducted a study where they created a scaffold made of freeze-dried gelatin, which was incorporated with MSCs and bFGFs. They implanted this scaffold in a rat model and observed that it increased the regeneration of epithelial tissue and chondrogenesis with less luminal narrowing and lower inflammation in the damaged region of the trachea. However, this was only observed for minor wound injuries (Özpolat et al., 2013).

##### 5.2.1.5 Hyaluronic acid and fibrinogen-based scaffold

Natural polymers such as Hylaograft C (Hyaluronic acid-based) and fibrin have also been used to engineer tracheal tissue. Weidenbecher et al. implanted a Hylaograft C scaffold seeded with chondrocytes into adult male rabbits for laryngotracheal reconstruction. Histological and endoscopic studies revealed a slow scaffold resorption, neocartilage formation with GAGs deposition, but an immature matrix, persistent scaffold remnants, and mechanical inadequacy for airway support led to graft failure (Weidenbecher et al., 2009). Fibrin-based scaffolds promote cell growth and proliferation by binding active biological molecules, enhancing cell-matrix connections and tissue regeneration. Heikal et al. created a fibrin-fibroblast bilayer construct coated with titanium mesh seeded with respiratory epithelial cells and implanted it into a sheep for tracheal epithelial regenesis. After 4 weeks of implantation, the researchers observed tracheal mucosal regeneration with minimal luminal stenosis and fibrosis through histological and fluorescence studies (Mohd et al., 2010).

#### 5.2.2 Synthetic scaffold

Synthetic polymers are produced under controlled laboratory circumstances and may be customized to possess precise mechanical characteristics, such as rigidity and flexibility, essential for maintaining the structural stability of the trachea and sustaining respiratory function. They provide significant benefits due to their ability to be readily modified to suit various specialized mechanical functions. However, their primary drawback is the lack of cell attachment and the need for chemical manipulation to increase cell adherence and function.

##### 5.2.2.1 Polycaprolactone

**FIGURE 6:**
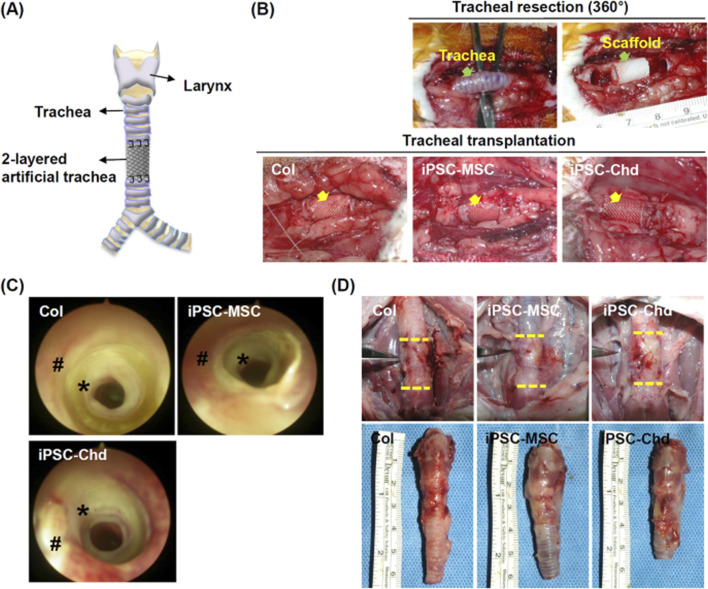
The Figure illustrates the process of tracheal transplantation and the subsequent analysis using an endoscope. **(A)** Diagram illustrating the placement of two-layered 3D bioprinted tracheal scaffolds in damaged sections of a rabbit’s trachea. **(B)** Complete insertion of artificial tracheal scaffolds into specific sections of the trachea that have problems. **(C)** Endoscopic pictures were taken after 4 weeks. All three groups had excellent maintenance of airway patency. **(D)** Macroscopic observations of the implantation site 4 weeks after surgery. Macroscopic observation of the collection of tracheal tissue (Kim I. G. et al., 2020).

PCL is a synthetic polymer with promising properties such as greater mechanical strength and a slower degradation rate than other biodegradable polymers. In animal models, the combination of PCL and sodium alginate hydrogels increased the development of respiratory ciliated cells, vascularization, and cartilage formation (Bae et al., 2018). According to a research done by Lin et al., a freeze-dried gelatin and collagen-based PCL scaffold was implanted in a mice model. The results showed that the grafts could regenerate tissue and mucosal lining completely. However, they lacked granulation formation and mechanical strength, which eventually led to graft rejection (Lin et al., 2008; Lin et al., 2009). Kwon et al. conducted a study using an asymmetric porous membrane made using Pluronic F127 and PCL to repair a rabbit tracheal defect in the anterior wall. They observed a normal lumen diameter of approximately 70% of the tracheal length after 12 weeks of transplantation. Based on their findings, they concluded that porous PCL could aid in promoting epithelial regeneration and airway patency. However, their conclusions were limited by the short lifespan of the graft and the constriction of the anastomosis site (Kwon et al., 2014). Kim et al. (2020) utilized electrospinning and 3D printing techniques to create a two-layered artificial tracheal scaffold composed of PCL. The outer layer of the scaffold was seeded with iPSC-derived MSCs and iPSC-MSCs-derived chondrocytes, while human bronchial epithelial cells were seeded in the inner regions. Following scaffold fabrication, they cultured it in a bioreactor system for 2 days before implanting it into a rabbit model with a segmental tracheal defect. Four weeks post-implantation, they observed the formation of slight granulation with ciliated columnar epithelium and neo-cartilage formation at the defect site. Figure 6 depicts the transplantation process of two-layered 3D-bioprinted tracheal scaffolds into damaged regions of the rabbit trachea, followed by their endoscopic and macroscopic assessment 4 weeks post-implantation (Kim I. G. et al., 2020).

##### 5.2.2.2 Polyglycolic acid

The idea of tracheal regeneration using tissue engineering approaches was introduced by Vacanti et al. He fabricated and implanted a chondrocyte-seeded polyglycolic acid (PGA) based non-woven mesh-coated silastic tube scaffold in nude mice, resulting in neo-tissue regeneration. However, when the tube was withdrawn, all animals died within a week after experiencing respiratory distress (Vacanti et al., 1994). In tissue engineering, PGA provides flexibility regarding degradation rates and porosity, promoting vascularization and ECM formation through cell-polymer interactions, and promoting cell growth (Bogan et al., 2016). Sakata et al. implanted a PGA mesh-coated scaffold seeded with epithelial cells in mice. They observed that neovascularized tissue had inflammation, whereas the epithelial lining on regenerated tracheal tissue was intact (Dhasmana et al., 2020). Fuchs et al. and their colleagues found that prenatal tracheoplasty with an engineered cartilage construct can be an effective therapy for severe congenital tracheal abnormalities, but it has limited application in adults (Fuchs et al., 2002). Subsequently, Luo et al. fabricated the PGA mesh non-woven wrapped with a silicone stent, and implanted it in rabbits. They observed the complete regeneration of tracheal tissue segments within 2 months of implantation. However, only 60% of rabbits have a survival period of more than 6 months after post-operation due to inflammation and incomplete mucosal formation (Luo et al., 2013). Gimmer et al. conducted an experiment where they implanted PGA and alginate-based scaffolds containing chondrocytes in rabbits. However, they observed a high mortality rate in animals undergoing tracheal reconstruction using PGA constructs due to respiratory complications. They also noticed the formation of mature tracheal cartilage at the implantation site (Grimmer et al., 2004). In a research study by Kojima et al., PGA, hydrogel, and PCL-incorporated chondrocytes were compared by implanting them into immunodeficient mouse models. The study found that PGA-based chondrocytes seeded on a scaffold exhibit better *in vitro* and *in vivo* results than using a hydrogel and a PCL-based scaffold for chondrocytes. Based on these findings, it can be concluded that PGA is a suitable material for tracheal tissue regeneration with few complications, such as incomplete vascularization, mechanical instability of the graft. Further investigation is needed as the % of survivors is a grave matter of concern in most experiments (Kojima and Vacanti, 2013).

##### 5.2.2.3 Poly(lactic-co-glycolic) acid

Poly-lactic-glycolic acid (PLGA) is a more easily applied biocompatible scaffold material that is promising for tracheal tissue engineering compared to other synthetic means. Lee et al. found that a rabbit tracheal wall (5 × 5 mm) can be replaced using an implanted PLGA scaffold seeded with cells, and noticed epithelialization at the site of excision, but were unable to identify viable chondrocytes in the implants (Lee et al., 2002). Tatekawa et al. implanted a PLGA/collagen-based scaffold seeded with bFGFs in a rabbit model. After 6 months, they observed improved mechanical strength but poor tracheal cartilage formation *in vivo* (Tatekawa et al., 2010). Tsao et al. used a solvent-casting technique to create a PLGA scaffold with stem cells and chondrocytes for tissue regeneration. After 4 weeks, the scaffold degraded more quickly and showed deformation, inadequate vascularization, low mechanical stability, mild chronic inflammation, and moderate airway patency (Tsao et al., 2014). Additionally, Wu et al. created a trilayer graft using PLGA, PLCL, collagen, and silk fibres. The graft was incorporated with smooth muscle cells and human umbilical vein endothelial cells (HUVECs) and implanted into a mice model. After 10 weeks of *in vivo* implantation, the graft exhibited significant cell proliferation, collagen matrix formation, cellular infiltration, and biodegradation, but had few drawbacks, such as a lack of layer-specific biomechanical properties and biocompatibility (Wu et al., 2018).

##### 5.2.2.4 Polypropylene

Polypropylene (PP) is a non-absorbable polymer commonly used in medical therapies. For trachea tissue engineering, implanting PP into the human body causes tissue degradation and graft rejection (Dhasmana et al., 2020). Several attempts have been made to design a PP-based scaffold without graft rejection. In a study, Kanzaki et al. (2006) designed a graft made of PP and Dacron, which was seeded with epithelial cells. This graft was implanted subcutaneously into white rabbits to regenerate a functional tracheal epithelial lining. After 4 weeks, they observed a fully functional epithelial lining on the bioartificial tracheal graft without a mucosal lining, difficulty in maintaining airway patency, and chronic inflammation (Kanzaki et al., 2006). Okumus et al. designed a prefabricated composite graft made of PP and epithelial tissues using a silicone catheter tube in another study. They implanted this axial biosynthetic graft into adult female rabbit models to reconstruct circumferential tracheal defects. After 4 weeks of implantation, histological, macroscopical, and radiological examinations showed neo-cartilage regeneration with mechanically stable properties and a complete mucosal lining. They also observed the vascular supply, with a lack of mucociliary functions and respiratory complications (Okumuş et al., 2005). In 2008, Tada et al. conducted a study on rats to regenerate tracheal epithelium using a scaffold made of PP-mesh coated with collagen sponge and vitrigel membrane. After 7, 14, and 28 days of implantation, they observed epithelium with a basal cell layer and columnar ciliated epithelium in the vitrigel model through histological examinations they failed to assess long-term airway patency, mucociliary clearance, lack of rigidity, and vascularization, needed for clinical translation (Tada et al., 2008).

##### 5.2.2.5 Polyurethane

Polyurethane (PU) is a synthetic polymer that exhibits excellent biocompatible, physical, and mechanical properties and also supports cartilage tissue growth in the trachea (Hsieh et al., 2018). Various studies have been conducted to regenerate the trachea cartilage tissue. In 2016, Jung et al. created PU-based cell-free tracheal scaffolds using a 3D printing technique and implanted them into rabbits. They discovered that the scaffolds could promote re-epithelization and connective tissue ingrowth with microvasculature through histological examinations. However, further research is required to confirm that PU can provide suitable tracheal biomechanical functions without cartilage regeneration over the long term (Jung S. Y. et al., 2016). In 2015, Clark and his colleagues created a tracheal graft by vacuum seeding a nanofibrous scaffold made of PU and polyethylene terephthalate (PET) with bone marrow mononuclear cells (BM-MNCs) and implanted it into the trachea of juvenile sheep. The study found that epithelial migration, wound healing, and neo-tissue formation occurred at the implantation site, with delayed stenosis for up to 6 weeks (Clark et al., 2016). Best et al. developed a tracheal scaffold using PU, PET, and polycarbonate materials through electrospinning and 3D printing techniques and seeded the scaffold with ovine bone marrow mononuclear cells (BM-MNCs). The scaffold was then implanted into the trachea of juvenile sheep to simulate the biomechanical characteristics of the native sheep trachea. They found that the solid C-shaped polycarbonate-based 3D printed rings show better cell seeding efficiency and regenerated tissue similar to the native trachea compared to porous ringed polyurethane and polyethylene terephthalate (PU/PET) scaffold (Clark et al., 2016).

##### 5.2.2.6 Polydimethylsiloxane and other scaffolding biomaterials

**FIGURE 7:**
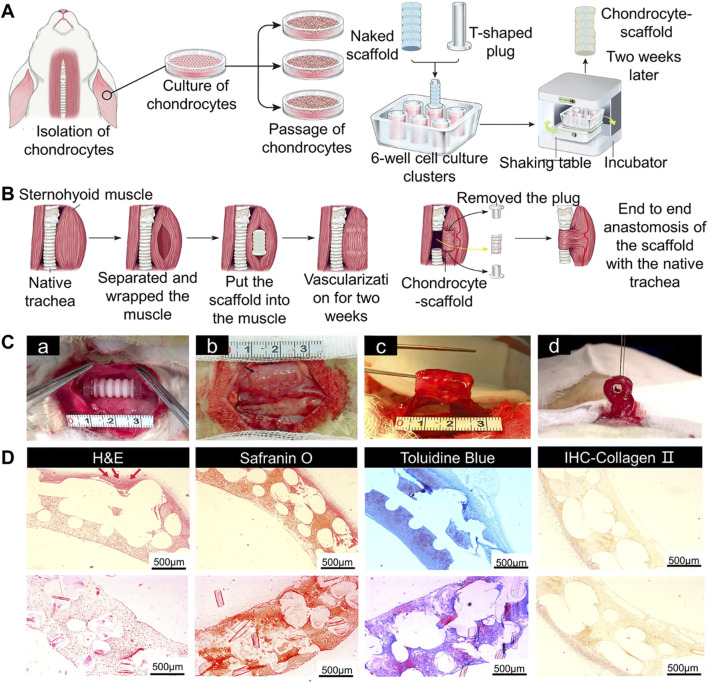
Tracheal cartilage scaffolds were made by 3D printing technology and tested *in vivo*. **(A)** Schematic diagram of a 3D printed PLLA tracheal scaffold. **(B)** Pre-vascularization and tracheal reconstruction of cellular scaffold constructs *in vivo*. **(C)** The scaffold was implanted into a sternal muscle for pre-vascularization. A complete segmental tracheal organ unit with a pedicle muscle flap was formed. **(D)** The images of H&E, safranin O, toluidine blue staining, and immunohistochemistry (IHC) of type II collagen of chondrocyte-scaffold constructs. (Scale bar: 500 μm; red arrow: small blood vessels around the engineered trachea (Wang et al., 2022).

Other synthetic biomaterials, such as polydimethylsiloxane (PDMS), have also been used to fabricate tracheal reconstruction. In another study, Poon (2018) created PDMS-based micro-engineered scaffolds with various biophysical and biochemical properties, such as topography and stiffness, to examine their impact on bio-functional tracheal regeneration. They discovered that a PDMS matrix with an alveolar-mimetic structure shows better cell viability and differentiation marker expression *in vitro* than collagen vitrigel membranes (Poon, 2018). Gao et al. (2019) developed a 3D printed PLLA-based scaffold designed to mimic the morphology of the rabbit trachea for segmental reconstruction. The scaffold was initially seeded with rabbit autologous chondrocytes and cultured *in vitro* for 2 weeks. Subsequently, it was transplanted into the muscular flap near the rabbit’s trachea to promote *in vivo* pre-vascularization and cartilage maturation for an additional 2 weeks. This innovative approach demonstrated that combining chondrocyte pre-culturing and pre-vascularization effectively regenerated a tracheal segment substitute with a bionic structure and mechanical characteristics close to the rabbit’s native trachea. The workflow for producing 3D-printed PLLA tracheal scaffolds seeded with rabbit chondrocytes, followed by their *in vivo* pre-vascularization and implantation, and the microscopic assessment of the resulting engineered constructs is shown in Figure 7 (Wang et al., 2022).

#### 5.2.3 Hydrogel-based scaffold

Hydrogels have many applications, including tissue engineering and drug delivery. In tissue engineering, hydrogels are currently used as a scaffold to construct new tissues. For tracheal tissue engineering, methacrylate hydrogels have been shown to mimic the biomechanical properties of ovine neonatal trachea (Soriano et al., 2021). Meanwhile, fibrin, agarose, and collagen type-1 hydrogels have been found to promote vascularization and ciliated respiratory epithelium of the trachea (Kreimendahl et al., 2019). However, using hydrogels as a tracheal substitute remains limited, but preliminary studies indicate they could be promising. However, more research is required to confirm the feasibility and mechanical integrity of hydrogel-based constructs for tracheal tissue regeneration without complications (Soriano et al., 2021). Ruszymah et al. created a composite by combining high-density polyethylene (HDP), Pluronic F127 hydrogel, and human nasal septum chondrocytes and subcutaneously implanted them into athymic mice for tracheal reconstruction. After 8 weeks of implantation, new cartilage formation with inflammation, lack of mechanical durability, and incomplete mucosal regeneration were also observed (Ruszymah et al., 2005). Park et al. fabricated a 3D ring-shaped PCL-based silicone bellow scaffolds using indirect 3D printing and coated with a layer of decellularized tracheal mucosa extracellular matrix (ECM) hydrogels and human inferior turbinate mesenchymal stromal cell (hTMSC) sheets. They implanted this tracheal graft into rabbits. After 2 months, a pre-clinical study showed successful healing with complete re-epithelization, absence of cartilage regeneration, and unstable biomechanics of the graft at the site of tracheal graft implantation (Park et al., 2018). Chang et al. (2018) created a PCL/PEG hydrogel scaffold encapsulated with auricular chondrocytes and implanted into rabbits for tracheal cartilage reconstruction. The results showed retention of chondrogenic ability and neo-tracheal cartilage formation, but displayed insufficient mechanical properties after 4 weeks of *in vivo* transplantation (Chang et al., 2018). Kim S. H. et al. (2021) designed a methacrylate silk fibroin (Sil-MA) hydrogel using a digital light processing (DLP) 3D printer and encapsulated it with chondrocytes. They implanted this hydrogel into a rabbit model for tracheal cartilage regeneration. Histological staining and RT-PCR showed epithelium formation and the development of cartilage-like tissue around the transplanted hydrogel. However, the graft failed to replicate complex architectures that require epithelium-cartilage-vascular coordination due to simplified geometries and monocultures. The majority of hydrogel-based scaffolds are generated via the use of 3D bioprinting, which will be examined in detail in the 3D bioprinting section 4.3.3.

Biomaterials for tracheal bioengineering have progressed from early synthetic polymers such as PGA and PCL, which offer mechanical support and biodegradability but often cause inflammation or fail to replicate the native compliance of tracheal tissues (Tsao et al., 2014), to the development of hybrid systems that integrate decellularized ECM for enhanced bioactivity with synthetic polymers designed for precise C-ring architecture (Mahfouzi et al., 2021). These innovative combinations have enabled the development of vascularized and epithelialized constructs with sustained luminal patency in preclinical studies. Although PGA scaffolds seeded with chondrocytes have been successful in regenerating cartilage-like tissue *in vivo* (Baiguera et al., 2014a), and PCL bioinks have shown effective integration of cartilage without stenosis (Tsao et al., 2014; She et al., 2021). As standardization improves and cell–scaffold interactions become better optimized, these emerging biomaterials hold promising potential for clinically viable, off-the-shelf tracheal grafts that can address the mechanical weaknesses and immunogenicity of earlier designs, ultimately supporting reliable long-segment tracheal reconstruction.

### 5.3 Fabrication techniques

Tissue engineering techniques largely rely on using various biomaterial scaffolds and cultivating multiple cell types on the scaffold to generate an airway-like structure, while incorporating growth factors to support regeneration (Kim H. et al., 2021). Tissue engineering has shown significant potential across various tissue systems, leading to growing interest in its application to respiratory medicine, particularly tracheal replacement (Tan et al., 2023). Various tissue engineering strategies such as decellularization, electrospinning, freeze-drying, and 3D bioprinting, have shown promising potential in developing tracheal substitutes. However, no construct has yet succeeded in simultaneously achieving robust vascularization, mechanical stability, and complete re-epithelialization (Nashihah et al., 2023; Mohd et al., 2010). Nevertheless, the techniques explored so far hold substantial promise and may ultimately establish the future gold standard for tissue-engineered tracheal grafts (Hutmacher, 2001; Subia and Kundu, 2010; Wei et al., 2024).

#### 5.3.1 Decellularization

**FIGURE 8:**
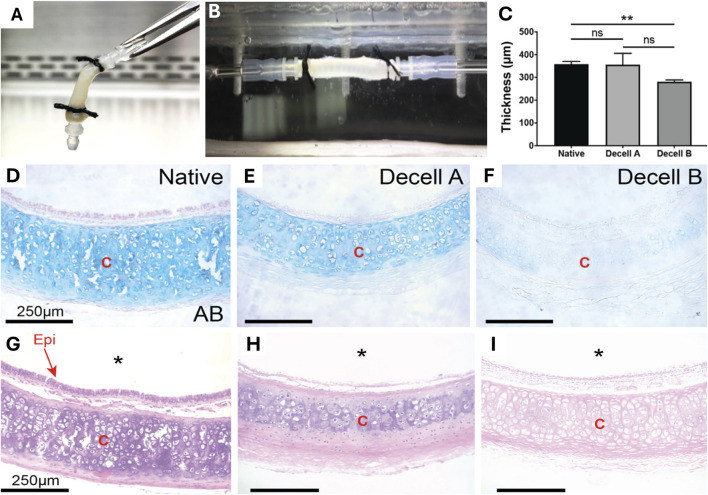
Mechanical and histological characterization of native and decellularized rat tracheas. **(A)** Macroscopic view of rat trachea post-decellularization and cannulation. **(B)** Image of a decellularized rat trachea positioned in a biaxial testing setup to assess mechanical properties. **(C)** Measured wall thickness of native, Decell A, and Decell B tracheal segments. **(D–F)** Alcian blue staining showing glycosaminoglycan content distribution in native, Decell A, and Decell B tracheas (Scale bar: 250 μm). **(G–I)** H&E staining demonstrating varying levels of cellular removal across native, Decell A, and Decell B tracheas (Scale bar: 250 μm) (“Epi” denotes epithelium, * denotes lumen, and “C” denotes cartilage) (Greaney et al., 2023).

**FIGURE 9:**
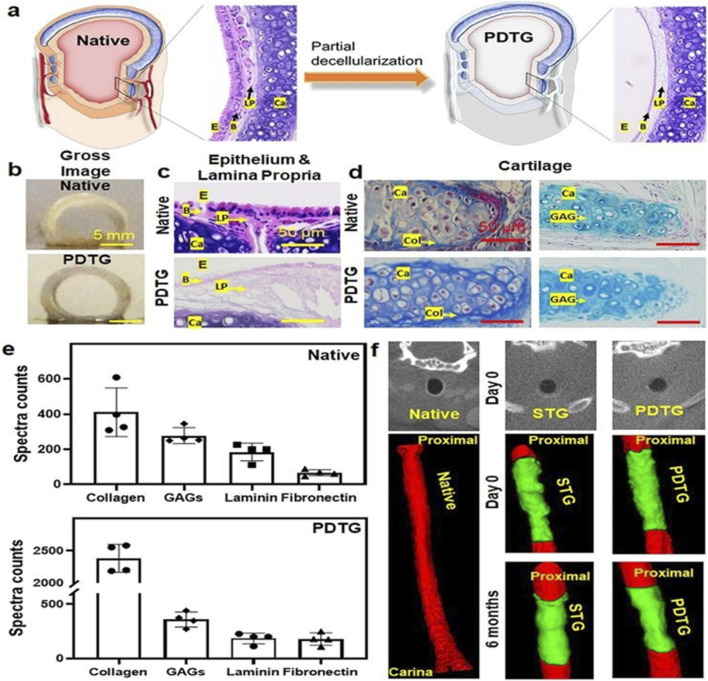
Characterization of a tracheal graft following partial decellularization. **(a)** Graphical illustration of the native trachea and the partially decellularized tracheal graft (PDTG). **(b, c)** Gross images and H and E staining of the native trachea and PDTG, showing the removal of all cells from the luminal surface of the epithelium and lamina propria through the partial decellularization process. **(d)** Masson’s trichrome and alcian blue staining of the tracheal cartilage, illustrating the preservation of extracellular matrix components using the partial decellularization process. **(e)** Mass spectrometric (LC-MS/MS) analysis of ECM proteins, including collagen, glycosaminoglycans, laminin, and fibronectin, in the native trachea and PDTG. **(f)** Micro-CT images of the native trachea, syngeneic tracheal graft (STG), and PDTG at day 0 and 6 months post-tracheal replacement (Tan et al., 2023).

One approach to the development of biological scaffolds for the regeneration of tissues and organs involves the use of decellularized matrices derived from humans, animals, or even plants. Decellularization is the process of removing cells from tissue, leaving behind the extracellular matrix (ECM) proteins that serve as a reservoir of essential growth factors and signaling molecules that play a critical role in regulating and maintaining tissue homeostasis, growth, and differentiation, as well as preserving the mechanical and bioactive properties of the tissue (Gupta et al., 2017). Decellularized tissue has received significant interest as a biological scaffold for tracheal bioengineering. In 2010, Baiguera et al. optimized a DEDM using SDC and DNase I on human tracheal tissue, achieving complete removal of nuclear and cellular components within 3 weeks. Histological and molecular analysis confirmed the preservation of the ECM and the mechanical properties of the tracheal scaffold. Importantly, immunohistochemistry and angiogenic assay showed that the acellular tracheal scaffolds retained the angiogenic factors, exhibiting chemotactic activity *in vitro*, and induced angiogenesis *in vivo* (Baiguera et al., 2010b). In 2012, Zang et al. established a decellularization protocol using SDC, NaCl, and DNase I to treat tracheal tissue from Brown Norway Rats. Histological and biochemical analysis confirmed complete antigen removal, preservation of the basement membrane, and significant loss of cartilage histoarchitecture and glycosaminoglycan (GAG) content. Mechanical testing showed reduced stiffness but retained adequate compressive strength to preserve tracheal lumen patency (Zang et al., 2013). In a 2013 study, Partington et al. developed a 25-cycle decellularization protocol using SDC and DNase I to process cadaveric pig tracheas. The method achieved complete decellularization of the submucosal region, although chondrocytes remained within the cartilage. Key extracellular matrix components such as fibronectin and laminin were preserved, while levels of soluble collagen, Type II collagen, and GAGs were notably reduced. Biomechanical properties, including tensile strength, declined progressively during the process, with significant reductions observed primarily at later stages (Partington et al., 2013). In a study by Shin et al., implanting chondrocytes seeded onto decellularized porcine cartilage scaffolds in a rabbit model led to the regeneration of respiratory tissue and neo-cartilage with a low inflammatory response (Kwon et al., 2014). In 2014, Baiguera et al. developed a decellularization protocol for rat trachea using SDC and DNase I within a bioreactor system. The resulting scaffolds were crosslinked with genipin to improve mechanical strength. After six cycles, the process effectively removed cellular content while preserving key structural and biochemical contents of the native tracheal ECM, including collagen, elastic and reticular fibers, and the luminal basement membrane. Mechanical properties were only slightly affected, with a minor, non-significant decrease in elastin, and genipin cross-linking helped partially restore tissue strength. Overall, the study demonstrated that bioreactor-based decellularization is a promising method for generating functional tracheal scaffolds, though it requires careful optimization to avoid damaging native tissue integrity (Baiguera et al., 2014b). In 2016, Sun et al. evaluated genipin-crosslinked decellularized rabbit tracheal scaffolds for their mechanical strength, angiogenic potential, and biocompatibility. Genipin treatment significantly increased the secant modulus, while CAM assays confirmed strong angiogenic responses in both treated and untreated scaffolds. In xenograft models, genipin-crosslinked scaffolds showed reduced inflammation over 30 days without elevating IgG or IgM levels. The study highlights genipin’s ability to enhance mechanical properties without compromising angiogenesis or immune tolerance (Sun et al., 2016). Chen et al. conducted a preliminary study in which they seeded respiratory fibroblasts and human bronchial epithelial cells into a decellularized scaffold using the air-liquid interface (ALI) method. The cells were left to grow for 3 weeks. These recellularized grafts were pre-vascularized in a rabbit model under *in vivo* conditions, leading to quicker tissue regeneration and improved cartilage regeneration (Chen and Kaji, 2017). Butler et al. discovered that decellularization of tracheal grafts using Triton X-100, SDC, RNase, DNase I, and a vacuum-assisted technique improves their quality for therapeutic applications. Acellular grafts have also been studied clinically for human tracheal tissue engineering (Butler et al., 2017). While this method required 9 days for complete decellularization, Hong et al. achieved comparable results in just 3 days and 12 h (Hong et al., 2018). Their protocol utilized a combination of hypertonic and hypotonic solutions, surfactants, protease inhibitors, peracetic acid, RNase, DNase, and thorough phosphate buffer saline (PBS) washes. Post-decellularization, the tracheal tissue showed a significant reduction in DNA content while retaining glycosaminoglycan levels and structural integrity. Cellular components were effectively removed without altering tensile strength or surface ultrastructure. Biocompatibility was confirmed, with no cytotoxic effects observed in contact assays. In 2018, Tchoukalova et al. assessed various decellularization protocols against the standard detergent enzymatic method (DEM) with agitation. The first experiment compared DEM, sonication, and lyophilization with freeze-thaw cycles, while the second tested a shortened SDC-based DEM against bioreactor-based decellularization. DNA levels dropped significantly after two cycles in all first-experiment groups, falling below 50 ng/mg by four cycles. However, residual nuclei in cartilage and MHC-1 staining in the submucosal region indicated potential immunogenicity. In the second experiment, DNA remained above threshold after four cycles, with stable collagen but significant GAG loss (Tchoukalova et al., 2017). In 2019, Giraldo-Gomez et al. introduced a novel decellularization technique using an ultrasonic bath and sequential chemical treatments, including Trypsin-EDTA, guanidine, SDC, Sodium dodecyl sulfate (SDS), tributyl phosphate, followed by a 72-h freeze-thaw cycle. Histological and molecular analyses (IHC and DAPI) confirmed effective removal of cellular material. Scanning Electron Microscopy (SEM) and thermal analysis showed the ECM structure was preserved, and biomechanical tests revealed no significant loss in mechanical strength. This method successfully produced a non-immunogenic tracheal scaffold suitable for *in vivo* applications. Recellularization showed early differentiation of stem cells into chondrocytes via SOX9 expression, highlighting its potential for airway regeneration (Giraldo-Gomez et al., 2019). In 2019, Batioglu-Karaaltin et al. explored the effects of combining lyophilization with various DEM agents, including deoxycholic acid, SDS, Triton X-100, DNase, and MgSO_4_, to evaluate the tracheal matrix’s structural integrity, biocompatibility, and composition while aiming to shorten the decellularization process to 3 days. Their results demonstrated that freeze-dried tracheal tissues treated with deoxycholic acid and DNase effectively eliminated cellular components while preserving GAG content and biomechanical strength, closely resembling native tissue. Additionally, cell viability assays confirmed the scaffold’s cytocompatibility (Batioglu-Karaaltin et al., 2019). In 2023, Greaney et al. employed two decellularization protocols, decell A and decell B on rat tracheas. Decell A involved perfusion with antibiotics, Triton X-100, NaCl, SDC, benzonase, and PBS, which effectively removed stromal and epithelial cells while preserving cartilage. Decell B used repeated treatments with SDC, DNase-I, and NaCl, thoroughly eliminating all cellular components, including chondrocytes. Decell A retained collagen I but lost collagen III, leading to increased scaffold stiffness compared to the native trachea. This shift reduced both circumferential and axial flexibility, with axial mechanics particularly impacted post-decellularization (Greaney et al., 2023) (Figure 8). Currently, most researchers are shifting their focus to partial decellularization procedures due to the limitations of complete decellularization. The complete decellularization process often results in high scaffold degradation rates, reduced retention of ECM components, loss of structural integrity and mechanical properties, and impaired vascularization (Tan et al., 2023; Dang et al., 2021). A recent study by Tan et al. demonstrates that partially decellularized tracheal grafts (PDTG) facilitate host-derived tracheal neo-tissue regeneration. Using a combination of microsurgical, transcriptomic, and immunofluorescent techniques, the study compared PDTG neo-tissue with surgical controls and native trachea. The results showed that PDTG neo-tissue, containing native airway cell types, exhibited microvasculature and neo-epithelium for at least 6 months. Within 2 weeks, PDTG achieved vascular perfusion, leading to the recruitment of multipotent airway stem cells with normal proliferation and differentiation properties. Consequently, PDTG neo-tissue replicates the architecture and functions of the native trachea and is capable of regeneration (Figure 9) (Tan et al., 2023).

#### 5.3.2 Electrospinning and other conventional methods

Several traditional techniques, including electrospinning, solvent casting, phase separation, and freeze drying, may be used to develop scaffolds for tracheal regeneration. The electrospinning process utilizes two distinct electrodes to create an electric field. This electric field creates ultra-thin strings with a high surface area, resulting in a porous structure. Due to this unique structure, such materials provide an ideal environment for cell growth and the formation of new tissue (Serrano-Aroca et al., 2022). Jang et al. developed a scaffold made of PCL/collagen using electrospinning. The scaffold was further modified with human umbilical cord serum. After implanting this scaffold in rat models with anterior tracheal defects, the researchers observed complete regeneration of tracheal cartilage and luminal epithelization, with less mechanical support and short-term mucociliary function (Jang et al., 2014). Interestingly, no inflammatory response was observed *in vivo* (Jang et al., 2014). In another study, researchers developed a nanofibrous scaffold using a combination of polyurethane and polyethylene terephthalate (PU/PET) through electrospinning. The scaffold was then seeded with autologous MSCs (mesenchymal stem cells) and placed in a bioreactor. Following this, the researchers have observed the formation of a fully regenerated vascularized tissue with mucosal and epithelial layers in a rat model. A recent study conducted involved the creation of a two-layered tubular biosynthetic scaffold using a combination of poly(L-lactide-co-caprolactone) (P(LLA-CL) and collagen fibers via an electrospinning technique. The scaffold was evaluated in a rat model with a tracheal wound. *In vitro* studies indicated that the graft’s microporous structure promotes pre-vascularization and cell proliferation. As a result, pre-vascularized grafts boost wound healing by forming new capillaries, reducing immunogenic response, and improving the regeneration of tracheal tissue, but there is a lack of evaluation of long-term patency and mucociliary function in implanted tracheal grafts were observed (Wu et al., 2017).

#### 5.3.3 Additive manufacturing and 3D bioprinting

**FIGURE 10:**
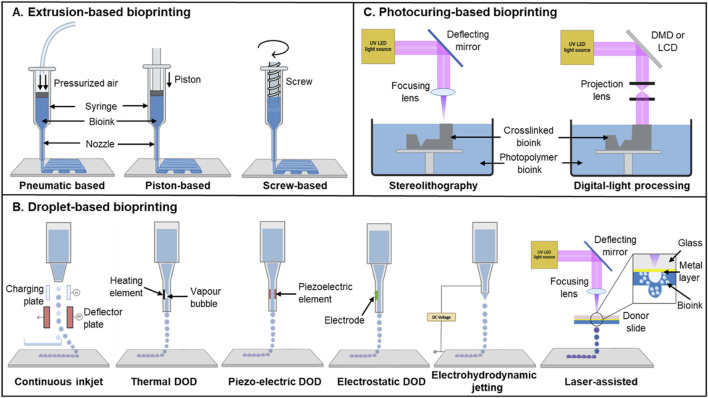
A cartoon representation of various 3D bioprinting strategies. **(A)** Extrusion-based bioprinting, which operates through pneumatic, piston-driven, or screw-assisted techniques. **(B)** Droplet-based bioprinting, encompassing techniques such as continuous-inkjet, thermal, piezo-electric, and electrostatic drop-on-demand, electrohydrodynamic jetting, as well as laser-assisted approaches. **(C)** Photocuring-based bioprinting, which uses digital-light processing and stereolithography techniques (Bisht et al., 2021; Kačarević et al., 2018; Huang et al., 2024).

Additive manufacturing (AM) is a fabrication technique that involves building objects by adding material layer by layer, as opposed to removing material in subtractive manufacturing (Bisht et al., 2021). AM encompasses a very intricate and comprehensive industrial production process, which includes the complete print workflow, where 3D printing functions as one component of the larger process, and 3D bioprinting forms a specialized subset of AM that uses cell-laden bioinks to create living tissue constructs with precise spatial control over heterogeneous cell types and bioactive cues. AM provides the engineering foundation that enables the biological sophistication of 3D bioprinting, with the key distinction being biocompatibility requirements, where AM focuses on mechanical precision and bioprinting requires high cell viability, shear-thinning rheology, and post-printing tissue maturation to bridge scalable manufacturing with regenerative medicine applications (Rothbauer et al., 2022). Advanced AM-based 3D printing approaches such as stereolithography (SLA), fused deposition modeling (FDM), selective laser sintering (SLS), Digital light processing, Poly Jet printing, extrusion-based, and droplet-based technologies have been widely used to create bio-scaffolds for tissue regeneration. Figure 10 shows a representative image of various 3D bioprinting techniques used to fabricate biomaterial-based scaffolds for tissue regeneration (Bisht et al., 2021; Kačarević et al., 2018; Huang et al., 2024). 3D bioprinting is an AM-based technique that uses live cells embedded in biomaterial filaments (bio-ink) and deposited layer by layer to fabricate synthetic prosthetic body parts. 3D bioprinting offers several benefits over various conventional methods, including the ability to print scaffold materials, bioactive components, and contour-fitting of cells in multiple layers in complex patterns. 3D bioprinting can achieve high precision control over porosity, pore size, and macro-morphology, thereby achieving proper control over the fabrication of the scaffold. 3D bioprinting can also help precisely deposit tissue growth factors, drugs, other biochemicals, and cells in the correct anatomic geometry during printing (Fishman et al., 2014; Zhang et al., 2021; Xia et al., 2018). Using bio ink deposition and the motorized stage movement, 3D bioprinting enables the creation of a 3D tissue construct using pre-programmed geometries and structures comprising living cells and biomaterials. This makes it ideal for designing hollow and complicated structures such as trachea and lung (Galliger et al., 2019). Biodegradable components such as PLA, PGA, PCL, and PLGA are used for 3D bioprinting, which has a strength similar to tracheal cartilage. As a result, several attempts are being made to create artificial tracheal constructs using 3D bioprinting technology (Chang et al., 2014).

Several research groups have attempted to use various tissue-engineered artificial tracheal substitutes to repair segmental tracheal defects. However, none have yet proven satisfactory for clinical use owing to airway stenosis (overgrowth of granulation tissue from the anastomosis site), airway collapse (softening and flattening of the framework), and mucus impaction (due to a lack of a respiratory epithelium) (Lee et al., 2016). In Table 3, we outline the various cell types and biomaterials used to generate artificial tracheal grafts for tracheal bioengineering via additive manufacturing and 3D bioprinting techniques, along with their respective outcomes. This summary offers a clear comparison of current bioprinting-based strategies employed in tracheal reconstruction. In 2014, Chang and colleagues designed a scaffold using extrusion-based bioprinting. The scaffold was made of PCL and coated with MSCs containing fibrin. The scaffold was implanted in rabbits with artificial tracheal defects. Histological and SEM-based examinations showed cilia regeneration, neo-cartilage formation, and less mechanical support without any rejection of the artificial tracheal graft (Chang et al., 2014). In 2015, Goldstein and colleagues created a scaffold of PCL and collagen gel seeded with mature chondrocytes using a 3D bioprinter and implanted it into New Zealand white rabbit models. Their histology and bronchoscopy observations noted tracheal cartilage regeneration and highly mucosalized tracheal lumen lining without tissue scarring or granulation. However, the functional validation of the graft remains preliminary and incomplete due to the short-term survival and integration of the graft (Goldstein et al., 2015). In 2015, Park et al. developed bellows grafts (artificial tracheal grafts) using a 3D bioprinting technique. The bellows graft comprised PCL and human turbinate mesenchymal stromal cells (hTMSC) sheets. The scaffold was then implanted in a rabbit model. After 4 weeks of implantation, the researchers observed the regeneration of tracheal epithelium along with highly ciliated epithelia. However, they did not perform biomechanical assessments to evaluate graft mechanics in comparison to those of the native trachea, nor did they assess circumferential mechanics or vascular requirements for segmental replacement (Park et al., 2015a). Park et al. 2015, implanted a 3D bioprinted PLCL/gelatin-based bellows scaffold seeded with human chondrocytes in nude mice, observed significant tracheal cartilage regeneration, and found mechanical properties similar to native trachea (Park et al., 2015b). Costantini et al. 2015, found that using alginate as a modeling agent in a 3D bioprinted scaffold, in combination with human bone marrow-derived MSCs (bMSCs) during the 3D bioprinting process, may improve the chondrogenic differentiation of bMSCs when cultured in chondrogenic medium. Moreover, combining cells with alginate leads to the creation of biomimetic inks well-suited for 3D bioprinting and cartilage tissue regeneration. Lack of mechanical testing does not confirm biomechanical match to native cartilage, and absence of long-term (>6 weeks) data omits hypertrophy reversal or stable phenotype maintenance (Costantini et al., 2016).

**TABLE 3:** A summary of the various cell types and biomaterials (both synthetic and natural) employed in tracheal tissue engineering through additive manufacturing and 3D bioprinting, along with their respective outcomes.

Cells	Biomaterials	Approaches	Outcomes	References
Mesenchymal stem cells (MSCs)	PCL	Extrusion-based 3D bioprinting	Epithelial cilia regeneration and neo-tracheal cartilage formation were observed	Wu et al. (2018)
Mature chondrocytes	PCL/Collagen	3D bioprinting	Well-mucosalized tracheal lumen lining and tracheal cartilage regeneration were observed	Hsieh et al. (2018)
Human turbinate mesenchymal stromal cells (hTMSC)	PCL (bellows grafts)	3D bioprinting	Tracheal epithelium regeneration, along with highly ciliated epithelia, was present	Jung S. Y. et al. (2016)
Human chondrocytes	PLCL-gelatin-based bellows scaffolds	3D bioprinting	Significant tracheal cartilage regeneration and mechanical properties similar to native trachea were seen	Clark et al. (2016)
Human bone-marrow-derived mesenchymal stem cells (bMSCs)	Alginate	3D bioprinting	Chondrogenic differentiation and tracheal cartilage tissue regeneration were observed	Poon (2018)
Reticular chondrocytes	PCL	Direct 3D bioprinting	Inflammatory reactions and tissue granulation, along with neo-cartilage formation	Bogan et al. (2016)
No cells	PCL and decellularized bovine dermal collagen matrix	3D-printing	Respiratory mucosa coverage and vascularization	Rehmani et al. (2017)
bMSCs	PCL	3D-printing	Enhanced biomechanical properties and slightly lower proliferation with higher early inflammatory response compared to decellularized tracheal tissues	Shan et al. (2017)
Human MSCs	PU	Direct 3D bioprinting	Chondrogenic differentiation and neo-cartilage formation (*in vivo* and *in vitro* studies) were observed	Yang et al. (2022)
Epithelial and MSCs	PCL	3D bioprinting	Chondrogenic differentiation and tracheal tissue regeneration were seen	Baiguera et al. (2010a)
No cells	PCL	3D-printing and electrospinning	Overgrowth of fibrous tissue and poor integration of the graft led to airway narrowing	Townsend et al. (2018)
Human MSCs	PCL	3D bioprinting	Smooth muscle, tracheal cartilage formation, and biomechanical characteristics similar to native trachea were observed	Chang et al. (2018)
Tracheal hyaline chondrocytes	PCL	3D bioprinting	New tracheal cartilage formation (intervening membrane) was observed	Kim S. H. et al. (2021)
Nasal epithelial cells and auricular chondrocytes	PCL/hydrogel	3D bioprinting	Epithelial cell regeneration and neonatal cartilage formation	Mahfouzi et al. (2021)
Autologous auricular chondrocytes	PCL	3D bioprinting	Epithelial-like tissue regeneration and better compressive strength than native trachea	Xia et al. (2019)
Human turbinate mesenchymal stromal cells	PCL-based silicone ring-shaped scaffold	Extrusion-based 3D bioprinting	Complete re-epithelialization was observed (histopathological analysis)	Baiguera et al. (2014a)
Human nasal chondrocytes (hNCs) and human nasal turbinate stem cells (hNTSCs)	PCL-based bellows framework	Two-step extrusion-based 3D bioprinting	Successful tracheal cartilage formation was identified	She et al. (2021)
Human MSCs	PCL/CNC (inner layer), nano-cellulose composite (outer layer)	3D bioprinting (inner layer) and electrospinning (outer layer)	Regeneration of epithelial tissue and tracheal cartilage formation	Kim H. et al. (2021)
Chondrocyte (C-rings), fibroblast (VF-rings)	Methacryloyl-modified gelatin, cartilage acellular matrix (C-rings), and methacryloyl-modified derm acellular matrix, 8-arm polyethylene glycol- succinic acid ester (VF-rings)	3D bioprinting	Formation of cartilage-vascularized fibrous tissue-integrated trachea (CVFIT) and mechanical properties similar to the trachea were observed	Tan et al. (2023)
Auricular chondrocytes	PCL and Sil-MA	Hybrid 3D bioprinting (FDM and DLP)	Improved mechanical strength and fracture resistance, promotion of fibroblast proliferation, accelerated tissue regeneration and vascularization, and maintenance of airway patency without triggering inflammation or stenosis	Lee et al. (2024)
Auricular chondrocytes and fibroblasts	GleMa, CSMA, and ElaMA	3D bioprinting	Epithelialization and complete vascularization throughout the c-shaped tubular cartilage rings	Nashihah et al. (2023)

In 2017, Kaye et al. demonstrated that using a tracheomalacia *ex vivo* model could replicate airway collapse, and a 3D printed external tracheal splint could effectively treat this collapse (Kaye et al., 2017). Shieh et al. 2017, explored 3D printing to create external airway splints for tracheomalacia, highlighting the potential for personalized medical devices in tracheal applications (Shieh and Jennings, 2017). In 2017, Gao and colleagues used a direct 3D bioprinting approach to create a biodegradable PCL-based scaffold seeded with reticular chondrocytes and implanted the scaffold into nude mice. After implantation, they observed neo-cartilage formation, tissue granulation, inflammatory reactions, and along with lack of pre-vascularization and epithelization of the implanted tracheal graft (Gao et al., 2017). Rehmani et al. 2017, designed 3D printed bioengineered tracheal grafts (BETGs) composed of PCL and ECM for reconstructing tracheal anterior defects in a porcine (large animal model). These grafts showed accurate anatomical fit, structural integrity, integrated well with native tracheal tissue, and supported re-epithelization and vascularization. Notably, five of seven animals survived the 3-month study with normal growth, confirming the biocompatibility and feasibility of BETGs for tracheal repair (Rehmani et al., 2017). In 2017, Shan et al. developed 3D-printed tracheal grafts using PCL and seeded with bMSCs to evaluate their biocompatibility and biomechanical properties. The bMSCs adhered, proliferated, and remained viable for 21 days without cytotoxic effects. Mechanical testing indicated that the grafts had significantly higher maximum stress and elastic modulus than native tissue, making them suitable for physiological airway pressures. *In vivo* studies showed good cell integration, despite an initial inflammatory response, highlighting the graft’s structural performance and cellular compatibility (Shan et al., 2017).

Taniguchi et al. (2018b), utilized a bio-3D printing approach to create scaffold-free artificial trachea-like constructs using MSCs, endothelial cells, and chondrocytes and implanted them in the F344 rat model. After transplantation, histological examinations showed vascularization and chondrogenesis, but the absence of an epithelial lining results in a lack of mucociliary function. In 2018, Hsieh and colleagues designed a tracheal construct using 3D printed PUs seeded with human MSCs through direct bioprinting. The construct was then implanted in nude mice. After 6 weeks, in both *in vivo* and *in vitro* studies, neo-cartilage formation and chondrogenic differentiation were observed, but there was a lack of dynamic airway patency due to the absence of an epithelial lining (Hsieh et al., 2018). Bae et al. (2018), conducted a study where they created a 3D bioprinted PCL-based scaffold, seeded with epithelial cells and MSCs. They studied the impact of growing MSCs in the standard media or chondrogenic differentiation media before growing them in the scaffold. After *in vivo* implantation, they observed that the scaffold seeded with MSCs that underwent chondrogenic differentiation exhibited a more significant potential for tracheal tissue regeneration. In 2018, Townsend et al. engineered a bioresorbable tracheal graft using 3D printed PCL rings with electrospun PCL nanofibers to provide structural support and air-tight sealing. The design aimed to preserve airway patency and enhance tissue integration during healing. In an ovine model, all animals survived for 10 weeks post-implantation. However, excessive fibrous tissue growth and poor integration led to airway narrowing. These findings highlight the need for improved degradation rates, enhanced cell adhesion, and antibacterial coatings (Townsend et al., 2018). Dongxu Ke et al. 2019, used 3D bioprinting to create PCL/hMSC-loaded tracheal scaffolds, which were analyzed using Western blot and immunohistochemistry. The study found evidence of smooth muscle and cartilage formation and significant biochemical similarities to native tissue (Ke et al., 2019). Kaye et al. 2019, designed a PCL scaffold and seeded it with tracheal hyaline chondrocytes using 3D bioprinting and implanted it in New Zealand white rabbits. The results of histopathology and intraluminal telescopic imaging showed that normal cartilage formation occurred at the implantation site when the chondrocytes were separated from the lumen of the trachea by an intervening membrane. If such a membrane is absent, stenosis and inflammation are more likely to occur (Kaye et al., 2019). In 2019, a study by Park et al. involved the creation of a PCL/hydrogel-based scaffold using a 3D bioprinter. The scaffold was seeded with auricular cartilage and nasal epithelial cells and ultimately implanted in a rabbit model. After 6–12 months, histological examination revealed epithelial cell regeneration, as well as neonatal cartilage formation with low mucociliary clearence (Park et al., 2019). In 2019, Xia et al. created a 3D bioprinted PCL-based tissue-engineered trachea (TET) using autologous auricular chondrocytes to imitate the goat trachea. The scaffold showed effective cellularization and better compressive strength than native tissue, confirmed by SEM and mechanical testing. In goat implants, the TET group had significantly higher survival rates than the control group with autologous grafts. While airway narrowing was noted in the TET group through bronchoscopy and CT, necrosis occurred only in the control group. Additionally, the TETs exhibited signs of epithelial-like tissue regeneration, suggesting their potential for clinical use (Xia et al., 2019).

**FIGURE 11:**
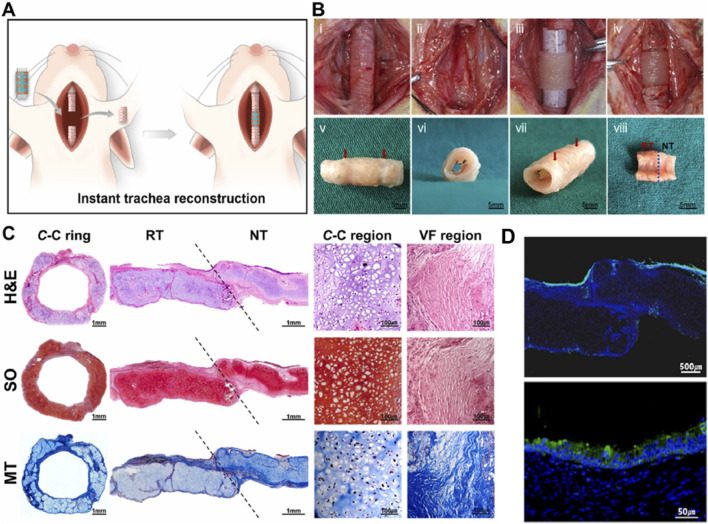
Instant reconstruction of the trachea using 3D bioprinted C-shaped tracheal biomimetic constructs. **(A)** Diagrammatic representation of repair of tracheal segment defects using direct end-to-end anastomosis. **(B)** (i–iv) images of direct end-to-end anastomosis surgical procedures used to repair the tracheal segment defects and (v–viii) gross images of reconstructed tracheal segments after 8 weeks of the surgery (Reconstructed trachea are represented by red arrows and the interface of regeneration between the NT and RT is represented by a blue dotted line) (Scale bar: 5 mm). **(C)** Histological staining, such as H&E (Hematoxylin and Eosin), SO (Safranin-O), and MT (Masson’s trichrome), is performed in both longitudinal and transverse sections of the reconstructed tracheal segments after 8 weeks of surgery (Scale bar: 1 mm and 100 μm). **(D)** Immunofluorescence staining of keratin (green) representing the tracheal epithelium regeneration after 8 weeks of surgery (Scale bar: 50 and 500 μm). Regenerated trachea: RT and native trachea: NT (Sun et al., 2024).

Lee J. Y. et al. (2020), created a PCL-based silicone ring-shaped scaffold using extrusion-based 3D bioprinting and seeded it with intact turbinate mesenchymal stromal cells (hTMSC), which was implanted in New Zealand white rabbits. After 17 weeks, histopathological analysis revealed full re-epithelialization. However, there was no cartilage regeneration, and the implanted tracheal graft lacked the biomechanical properties of the native trachea. Park et al. (2021), developed a two-step extrusion-based 3D bioprinting technique to create a trachea-mimetic cellular construct of clinically relevant size. The construct was tested in a mouse model and showed successful tracheal cartilage formation with lack of airway patency and mucociliary clearance. A group of researchers, Feng et al. 2022, developed a bilayer tubular scaffold for an artificial trachea. The scaffold is composed of PCL/CNC (cellulose nanocrystals) and was constructed using 3D printing for the inner layer and nano-cellulose composite using electrospinning for the outer layer. They introduced MSCs into the outer and inner layers and found that the bilayer tubular scaffold stimulated epithelial tissue and cartilage regeneration, fail to assess airway patency, mucociliary clearance, and vascularization (Feng et al., 2022). Huo et al. 2022, developed a strategy for the reconstruction of a fully functional trachea using cartilage-vascularized fibrous tissue-integrated trachea (CVFIT) via 3D bioprinting, which resulted in better mechanical functions and successful regeneration of trachea (Huo et al., 2022). In 2024, Lee et al. introduced a novel hybrid 3D bioprinting approach combining FDM with DLP to create an artificial trachea. PCL was printed via FDM for mechanical support, while chondrocyte-laden Sil-MA hydrogel was integrated using DLP. The resulting PCL-Sil scaffolds exhibited enhanced flexibility, fracture resistance, and supported fibroblast proliferation, indicating good biocompatibility. *In vivo* tests in a rabbit tracheal defect model showed effective tissue regeneration, with pre-culturing in the omentum improving vascularization. Eight weeks post-transplantation, bronchoscopy and histological analyses confirmed preserved airway patency without stenosis or inflammation. The hybrid technique also successfully printed trachea, vertebral bone disks, and trachea-lung structures (Lee et al., 2024). Sun et al. 2024, introduced an innovative technique for *in situ* tracheal reconstruction using 3D bioprinting combined with direct end-to-end anastomosis. Initially, tissue-specific matrix hydrogels were prepared, including GelMa crosslinked with chondroitin sulfate methacryloyl (CSMA) for cartilage-tissue-specific gels using a hybrid photo-crosslinking technique, and elastin methacryloyl (ElaMA) for fibrous-tissue-specific gels. These hydrogels were then mixed with chondrocytes and fibroblasts derived from the auricular cartilage of rabbits. Through 3D bioprinting, C-shaped tubular cartilage rings with vascularized fibrous tissue integrated into tracheal biomimetic constructs were created and implanted into nude mice. Eight weeks post-implantation, the reconstructed tracheal segments exhibited epithelialization and complete vascularization throughout the tubular structure. A schematic of instant tracheal reconstruction using a 3D-bioprinted C-shaped biomimetic tracheal construct, showing the entire process from defect creation to post-implantation evaluation, is depicted in Figure 11 (Sun et al., 2024).

To effectively portray the dynamic behavior of tissue regeneration, 3D printed tissues must include the element of time. To do this, scientists developed a technique called 4D bioprinting, which utilizes cell-adhesion forces and stimuli-responsive biomaterials to generate dynamic tissue architectures (Antezana et al., 2023). 4D bioprinting extends 3D bioprinting by incorporating the element of time, allowing printed constructs to change their shape, stiffness, or function in response to external stimuli such as temperature, pH, light, or biological signals. This approach utilizes smart hydrogels that are capable of controlled swelling, degradation, or cell-driven remodeling, thereby better replicating natural tissue development (Arif et al., 2022). Gao et al. defined “4D bioprinting” as the process of using 3D printing to create structures that include cells capable of responding to internal cell tensions or external stimuli (Gao et al., 2016). Kim et al. 2020 created an artificial trachea biomimetic tissue with silk fibroin methacrylate (Sil-MA) hydrogel sheets and human chondrocytes and turbinate-derived mesenchymal stem cells (TBSCs) using 4D-bioprinting and implanted it into a rabbit’s damaged trachea. After 8 weeks of implantation, the tracheal tissue construct merged with the host tracheal tissue naturally, and cartilage and epithelium were observed at the expected sites. This study demonstrated that 4D-bioprinting also creates a tissue biomimetic scaffold, highlighting its potential for tissue engineering and therapeutic applications (Kim S. H. et al., 2020).

Fabrication techniques have become a crucial aspect in tracheal bioengineering, significantly influencing a graft’s ability to mimic native shape and stiffness, while also enhancing its potential for effective cell seeding, vascular ingrowth, and host tissue integration. Multimodal strategies, such as combining 3D printing for macroscale geometry, electrospinning or lithography for microscale topography (Gao et al., 2017), and utilizing decellularized or bioink-based layers for biological guidance, have achieved greater patency and structural stability in preclinical models than earlier single-method constructs (Mammana et al., 2024). Despite advances in structural mimicry, engineered tracheal grafts still fall short of native function in several key areas. Mucociliary clearance remains poor, with PCL scaffolds exhibiting limited, disorganized ciliation and no glandular activity, resulting in mucus retention and an increased risk of infection (Lee et al., 2025). Mechanical performance is also inferior, as 3D-printed and electrospun constructs collapse more easily or develop fibrotic stenosis. Synthetic scaffolds frequently trigger foreign-body immune responses (Khalid et al., 2023; Seong et al., 2026), and even decellularized matrices cannot fully prevent persistent macrophage activity. Most critically, no engineered graft has achieved functional innervation, failing to reproduce trachealis contraction, cough reflexes, or electrophysiological responses (Wei et al., 2024). While vascularized pedicle techniques improve integration, they do not restore dynamic neural control (Wang Z. et al., 2025). Future progress will rely on multifunctional bioinks that incorporate neural and stromal progenitors, angiogenic and neurogenic factors, and bioreactors that replicate physiologic airway forces, as validated through robust large animal orthotopic studies. Collectively, advancing fabrication technologies and functional validation strategies will be essential for moving long-segment tracheal reconstruction toward reliable clinical translation (Wei et al., 2024).

## 6 Regulatory affairs in regenerative tracheal therapies

As interest in regenerative medicine continues to grow, so does the need to examine its regulatory framework. Definitions, terminology, categorization, and regulations related to cellular-based therapies vary widely across regions. This diversity creates a need for global unification to reduce heterogeneity and promote regulatory convergence for cell and gene therapy products. According to Chapter 1 of Article 2 (EC/1394/2007), Tissue-Engineered Products (TEPs) are defined as “a product that contains or consists of engineered cells or tissues and that can be used to regenerate, repair, or replace human tissue” (Joyce et al., 2023). Any gene therapy, somatic cell treatment, or tissue-engineered product is classified as an Advanced Therapeutic Medicinal Product (ATMP) and is regulated by EU Regulation (EC) No 1394/2007, which was established on 30 December 2008, in Europe. All ATMPs must comply with regulations and related medical laws to ensure quality, safety, effectiveness, as well as good manufacturing and clinical practices. In the United States (US), the Food and Drug Administration (FDA) oversees the regulation of medical devices, including TEPs and 3D bioprinted products. The data required to support a regulatory submission distinguishes these FDA regulatory classes as Biologics License Application (BLA), Premarket Approval, or 510(k), depending on the product designation. The FDA ensures that TEPs and additively manufactured products undergo thorough premarket evaluations concerning safety, effectiveness, and high quality, with an emphasis on biocompatibility, manufacturing practices, and clinical testing, particularly for applications such as tracheal implants. 3D bioprinting is an innovative technology that has the potential to revolutionize medicine by enabling the development of complex tissue structures, including tissue-engineered trachea. However, the promise it holds also means that the regulatory landscape is intricate and evolving, making the use of 3D bioprinting for critical applications, including tracheal implants, quite complex (Wei et al., 2024; Mirshafiei et al., 2024).

### 6.1 Biocompatibility, material safety and manufacturing standards

The biocompatibility of these materials is essential for obtaining regulatory approval for tissue-engineered products (TEPs) and 3D bioprinted products (Jain et al., 2024). It is crucial to conduct extensive testing to ensure that no adverse reactions occur once the implants are placed in the human body. For tracheal implants that are used for respiration, several important *in vitro* and *in vivo* tests are performed to assess how the materials interact with human tissues and their long term stability (Stehlik et al., 2024). Moreover, regulatory agencies require that 3D bioprinted tracheal implants be produced under strict quality control standards. This requirement ensures that both the design of the bioprinting and the manufacturing process consistently adhere to predefined specifications. It is vital to maintain the integrity of the bioprinting environment, ensure the quality of the inks and cells used in the bio-printing, and validate the overall production process.

### 6.2 Preclinical and clinical testing

Before regulatory authorities can approve a 3D bioprinted tracheal implant, thorough preclinical and early clinical tests must be conducted (Mammana et al., 2024). Initially, preclinical studies in animal models assess the implant’s applicability, safety, and potential risks. Following these, human clinical trials must be performed in several stages, starting with initial tests in humans and progressing to larger studies that evaluate safety, efficacy, and any associated side effects. All clinical studies should adhere to established protocols and regulations in accordance with the regulatory body’s approval of the implant (Walker et al., 2024). Regulatory pathways for tracheal implants remain largely undeveloped, with most work limited to compassionate-use cases, preclinical GMP evaluations, and early investigational studies rather than full regulatory approvals. This gap underscores the ongoing challenge of balancing urgent clinical needs with the demands of strict regulatory oversight (Sekar et al., 2021). One major concern is batch-to-batch variability from decellularized scaffolds and cell-laden bioinks. Factors such as donor tissue differences and variations in bioprinting can result in inconsistent mechanical and biochemical properties, necessitating extensive process validation in accordance with ICH Q9 principles (Kakroodi et al., 2025). In addition, huge cost estimates, often exceeding one to five million dollars per manufacturing campaign due to the need for GMP-compliant cleanroom facilities, specialized bioreactor systems, autologous cell expansion, and comprehensive Chemistry, Manufacturing, and Controls (CMC) documentation for Investigational New Drug (IND) submissions. Low economies of scale for rare conditions like long-segment tracheal stenosis further complicate this (Haeusner et al., 2021; Cossu et al., 2018; Kakroodi et al., 2025). GMP compliance also imposes strict requirements for aseptic processing, environmental monitoring, validated sterilization of bioinks, and complete traceability, which are particularly challenging in 3D bioprinting dynamic workflows involving living cells, frequently resulting in production delays, clinical holds, or reliance on hospital exemptions (EU Article 17) rather than full marketing authorization (Wang Z. et al., 2023; Joyce et al., 2023; Polak, 2010). Collectively, these barriers highlight the importance of adopting Quality-by-Design approaches, engaging in early regulatory consultations (e.g., FDA pre-IND meetings), and aligning with regulatory frameworks to facilitate the safe and effective translation of tracheal implants into routine clinical practice (Sekar et al., 2021). One of the earliest large-scale clinical efforts involved synthetic and decellularized tracheal scaffolds seeded with autologous stem cells, classified as ATMPs under EC No. 1394/2007, navigating hospital exemption via Article 17 that permits non-routine use without full marketing authorization when no satisfactory alternative exists. These constructs were produced under GMP conditions at Karolinska University Hospital, with validated decellularization achieving a DNA content of less than 50 ng/mg, and the absence of HLA class I/II expression. Sterility and endotoxin compliance were verified by MHRA/HTA licensing in the UK for compassionate implantation in a pediatric patient with long-segment stenosis. Preclinical qualification involved bioreactor maturation, vascular perfusion testing, and ethical approvals from the Swedish Medical Products Agency, but post-implant complications (stenosis, graft failure) triggered independent investigations revealing insufficient preclinical efficacy data and ethical lapses, halting further cases and emphasizing mandatory Phase I/II bridging studies (MacChiarini et al., 2004; Baiguera et al., 2010b). In the US, the laryngo-tracheal tissue-engineered transplant trial (NCT01997437) received FDA compassionate-use approval to evaluate hybrid decellularized matrices seeded with epithelial and chondrocyte populations for the treatment of severe airway defects. This pathway required an Investigational New Drug (IND) application with extensive CMC documentation covering decellularization validation, cell sourcing, and bioreactor protocols, along with large animal orthotopic studies demonstrating over 80% airway patency at 6 months and full IRB oversight, though patient recruitment remained difficult due to the rarity of eligible cases (Elliott et al., 2017). More recently, a 2025 collaboration between Michigan Medicine and Materialise earned FDA Breakthrough Device Designation for 3D-printed bioresorbable external tracheal splints for infant tracheobronchomalacia (TBM), expediting review through a 510(k) pathway by referencing predicates for absorbable airway stents. The designation was supported by ovine model data showing stable airway patency under ventilation pressures of 30–50 cm H_2_O, reduced granulation compared to metal stents, and validated manufacturing controls ensuring consistent PCL bioprinting and sterilization (Zopf et al., 2013). Preclinical regulatory efforts focus on GMP-compliant manufacturing for IND studies, exemplified by the pressurized transmural decellularization of porcine trachea, which meets MHRA standards for ATMP production. This process retains over 90% of collagen, maintains GAG levels above 70%, and exhibits mechanical properties similar to those of native trachea, with suture retention surpassing 10N, positioning scaffolds for future EU/US trials. Custom 3D-printed PCL tracheal grafts are pursuing FDA 510(k) clearance through comparisons to silicone and polyurethane stents, supported by ISO 10993 biocompatibility data, degradation kinetics (complete resorption 12–18 months), and finite element-validated mechanical performance under cough simulation (200 cm H_2_O), although scaling production from rabbit to porcine models remains a major CMC barrier. Collectively, these efforts signal a move toward harmonized regulatory frameworks such as ICH Q12, emphasizing quantitative patency metrics, long-term immunogenicity assessment, and post-market registries to progress from compassionate use toward routine clinical approval (Khalid et al., 2023; Sekar et al., 2021).

### 6.3 Ethical considerations

The ethical implications of large-scale 3D bioprinting, especially for complex organs like the trachea, are significant (Rostamani et al., 2024). These considerations include ethically sourcing cells, obtaining informed consent from patients, and understanding the long-term effects of bioprinted implants. Institutions should establish fair guidelines to weigh the benefits of 3D bioprinting against potential risks, ensuring that patients are fully informed.

### 6.4 Post-market surveillance

Monitoring should not cease once a 3D bioprinted tracheal implant is approved. Post-market surveillance is crucial for ensuring the long-term safety and effectiveness of these implants. This involves tracking patient outcomes, reporting any adverse events, and conducting periodic reviews to maintain standards in post-market follow-up. Continuous monitoring is essential to identify any potential issues.

### 6.5 International harmonization

Given the global nature of medical innovation, harmonizing international regulatory standards for 3D bioprinted medical products is imperative. Organizations within the International Medical Device Regulators Forum are actively working to align disparate regulatory requirements from various countries. Such harmonization simplifies the approval process and ensures the safety of tissue-engineered products (TEPs) and 3D bioprinted products for patients worldwide.

## 7 Challenges and future prospects in tracheal tissue engineering

The trachea is a structurally and functionally complex organ characterized by a diverse array of specialized cell types (Fang et al., 2020), intricate vascular networks (Salassa et al., 1977), robust biomechanical properties (Safshekan et al., 2016), and a finely tuned immune surveillance (Gozzi-Silva et al., 2021), maintaining effective respiratory function. Replicating these multifaceted attributes in engineered tracheal constructs remains a significant challenge (Gomes et al., 2025). Inadequate structural reinforcement, insufficient mechanical strength, poor biocompatibility, or lack of neo-vascularization can lead to deformation, compromised function, and eventual graft failure (Hollister et al., 2002). Advances in tissue engineering, including decellularization, synthetic scaffold development, and 3D bioprinting, have expanded the scope of clinical translation, albeit with their respective limitations (Adamo et al., 2022). Decellularized tracheal scaffolds leverage native ECM cues, and pre-clinical orthotopic models have demonstrated reduced immunogenicity, endothelial repopulation, restoration of near-physiological stiffness, re-epithelization, and improved luminal patency contingent upon adequate revascularization (Gomes et al., 2025). However, the clinical translation of these scaffolds remains limited due to protocol-dependent variability. Challenges such as residual antigens, weakening of cartilage segments, and partial recellularization can lead to graft collapse, stenosis, and inconsistent long-term performance, underscoring that native ECM alone does not ensure reliable regeneration (Lei et al., 2021; Liu et al., 2021; Nahumi et al., 2023). In contrast, synthetic tracheal grafts initially appeared promising due to their off-the-shelf availability and tunable mechanical properties; however, they consistently failed to function as viable living replacements (Greaney and Niklason, 2021). Numerous studies and histological reports indicate that non-vascularized polymer tubes, even when seeded with bone marrow-derived cells, often lead to infection, granulation, dehiscence, and ultimately patient death/further intervention, making them a paradigmatic example of how the lack of proper epithelia (mucociliary clearance) and microvascularization reliably contributes to the failure of synthetic airway constructs (Fux et al., 2020).

To address the limitations of conventional scaffold-based approaches, 3D bioprinting has recently emerged as a powerful strategy in tracheal tissue engineering. This technology enables the precise, layer-by-layer fabrication of complex macro- and micro-scale tissue architectures, allowing for the spatially controlled deposition of multiple cell types, biomaterials, growth factors, as well as the creation of cartilage-like rings and soft luminal compartments (Galliger et al., 2019; Khalid et al., 2023). Consequently, 3D bioprinted tracheal constructs in animal models can closely mimic native geometry, support chondrogenesis, and vascularized connective tissue formation (Gao et al., 2017; Wang et al., 2024). Notably, recent applications of 3D printed tracheal splints have advanced to compassionate clinical use, highlighting the translational potential of this technology (Zopf et al., 2013). Despite its promise, 3D bioprinting of the trachea poses several challenges that must be addressed to achieve viable functional tissue constructs. Notably, early developments in 4D‐bioprinted tracheal patches indicate promising potential for clinical application. One of the foremost hurdles is replicating the complex anatomical and biomechanical features of the native trachea. The printed scaffold must exhibit an appropriate elastic modulus to support natural cell proliferation and maintain airway patency after implantation (Hollinger et al., 1996). Achieving this requires constructing grafts that not only match the physical and mechanical properties of the native trachea at the macroscopic level but also support essential biological processes such as ECM remodeling, cell proliferation, and migration at the nano- and microscale for effective tissue integration (Hollister et al., 2002). Additionally, the scaffold must be engineered with biomechanical properties that can withstand physiological stresses and have a degradation rate that aligns with tissue regeneration for long-term functional integration (Mahfouzi et al., 2021).

Another essential aspect is the use and integration of multiple cell types essential for replicating native tracheal function (Mammana et al., 2024). Successful tracheal constructs require both structural cartilage, stromal layer and an inner epithelial lining generated from appropriate cells. However, co-printing these diverse cell types poses challenges, as conditions suitable for one cell type may compromise the viability or function of another. The spatial organization of cells within the printed scaffold is also crucial and demanding. Cartilage rings need to be precisely positioned for rigidity, while epithelial cells must form a continuous barrier to prevent infection and facilitate mucociliary clearance (Park et al., 2021). Differences in cell size, growth rates, and nutrient needs can lead to uneven cell distribution and loss of viability during or after bioprinting. Maintaining cell viability throughout the bioprinting process can be difficult due to mechanical stresses or damaging forces, particularly in extrusion printing (Zhang et al., 2024a). Immunological challenges may also arise with employing multiple allogeneic or poorly accepted cell types, increasing the risk of immune rejection and inflammation (Mammana et al., 2024). To address these issues, researchers are exploring dual-head or multi-material bioprinting to accurately deposit different bioinks with specific properties. Strategies such as combining thermoplastics like PCL for structural support with cell-friendly hydrogels for biological integration are gaining attention (Wei et al., 2024; Wang et al., 2024). Additionally, approaches like sequential bioprinting and co-seeding with iPSC-derived progenitors have demonstrated improved post-implantation survival and functionality (Kim I. G. et al., 2020).

Vascularization remains one of the most critical challenges in 3D bioprinted tracheal grafts. It is essential for delivering oxygen and nutrients, removing metabolic waste, and supporting the survival and functionality of embedded cells (Kaully et al., 2009). However, most bioprinted tracheal constructs lack a robust microvascular network, resulting in hypoxia, cell death, fibrosis, poor integration, or even graft failure due to ischemia. These issues provoke excessive immune responses and inflammation (Khalid et al., 2023; Shen et al., 2023). To address these factors, several vascularization strategies have been explored. Sacrificial bioinks can be used to create temporary channels that are subsequently removed, forming perfusable lumens suitable for endothelization (Cheng et al., 2023). Microfluidic-based bioprinting allows for the incorporation of branching microchannel networks that closely mimic native capillaries (Vera et al., 2021).

Additionally, the controlled release of angiogenic factors such as VEGF, bFGF, TGF-β, and angiopoietins, often delivered through encapsulated microparticles or tethered hydrogels, promotes rapid vascular ingrowth while minimizing systemic exposure (Liang et al., 2025). Further investigation into their deposition method and release kinetics is needed. Another effective strategy involves pre-seeding constructs with endothelial cells (e.g., HUVECs) alongside stromal or mesenchymal cells, or using pre-vascularized collagen and hybrid PCL–hydrogel composites, which can establish stable vascular networks in both *in vitro* and *in vivo* settings, thereby improving perfusion and reducing stenosis after implantation (Chen et al., 2021). *In vivo* pre-vascularization approaches, such as maturing grafts within well-vascularized muscle or platysma flaps before orthotopic implantation, further enhance anastomosis and mucosal regeneration, offering a promising translational pathway from experimental bioprints to clinically robust tracheal grafts (Lee M. et al., 2020; Liang et al., 2025; Zhang et al., 2024b).

The risk of graft rejection and adverse immunomodulatory responses also presents a significant challenge that affects both graft integration and long-term functionality. Studies have shown that common synthetic polymers like PLA and PCL are known to trigger acute and chronic inflammatory reactions upon implantation, leading to elevated levels of immunoglobulins (IgG, IgM) and pro-inflammatory cytokines such as IL-2 and IFN-γ, accompanied by cellular infiltration rich in eosinophils, indicative of a foreign body reaction (Panja et al., 2022; Wang Y. et al., 2023). Factors such as poor scaffold design, incomplete graft-host integration, and biological mismatch further amplify these immune responses (Wei et al., 2024). Unlike decellularized tracheal grafts, which retain components of the native ECM and are typically less immunogenic, synthetic 3D-printed scaffolds often lack immunoregulatory cues, making them prone to complications like graft stenosis or failure (Gomes et al., 2025). To combat these issues, researchers are exploring various immunomodulatory strategies. These include biofunctionalizing scaffolds with anti-inflammatory molecules, using autologous cells, and designing a hybrid scaffold that promotes quick vascular and epithelial integration (Barthes et al., 2021). Additionally, approaches like coating scaffolds with hydrogels, pre-seeding with immunoregulatory stem cells like iPSC-derived MSCs, and employing advanced bioprinting techniques that mimic the native trachea show promise in enhancing graft acceptance and long-term functionality (Kim I. G. et al., 2020).

Standardizing trachea-specific bioinks and their properties for 3D-bioprinted tracheal grafts remains challenging because replicating the trachea’s anisotropic mechanical structure, which involves rigid cartilage C-rings alternating with compliant and vascularized tissue, is complex, while maintaining high print resolution, cell viability, and long-term stability under cyclic airway pressures (Zhang et al., 2024a). Current bioinks exhibit significant variations in rheological properties, which can lead to issues such as poor filament fusion, shape loss, pore collapse, and mechanical mismatch, ultimately increasing the risk of luminal narrowing or graft failure (Theus et al., 2020). Strategies focus on establishing rheological and mechanical “windows” with specific ranges for shear-thinning, yield stress, viscoelastic moduli, and recovery profiles to enhance filament stability, ring geometry, and anisotropic stiffness, thereby mimicking the properties of native tracheal cartilage (Elango and Zamora-Ledezma, 2025). These must be paired with assays that link material properties to biological outcomes, such as cell viability, chondrogenic differentiation, and epithelialization (Huo et al., 2022). Emerging trachea-mimetic constructs generated using ECM bioinks, peptides, and hydrogel–polymer systems demonstrate how optimization strategies can guide formulations that maintain luminal patency while supporting chondrogenesis (Zheng et al., 2025). This paves the way for categorizing tracheal bioinks based on validated property ranges rather than qualitative descriptions. Ultimately, community standards for printability, reporting, and reference bioinks are crucial for achieving regulatory approval, ensuring reproducibility, and facilitating the clinical translation of 3D bioprinted tracheal grafts (Gao et al., 2025).

Amid the persistent challenges in tracheal tissue engineering, significant gaps exist between the native trachea and engineered constructs in structure, function, biomechanical properties, and stability, limiting their clinical application (Yeou and Shin, 2025).

The native trachea features a biological and biomechanical design, with hyaline cartilage C-rings providing robust resistance against airway collapse and a submucosal vascular network supporting mucociliary clearance and immune function (Furlow and Mathisen, 2018). These characteristics ensure mechanical durability, immune tolerance, and continuous self-remodelling (Safshekan et al., 2016; Hiemstra et al., 2015; Wei et al., 2024). In contrast, engineered constructs often fail to mimic this stability. Decellularized scaffolds lose glycosaminoglycans and cartilage integrity post-processing, making them prone to luminal stenosis, while residual DNA can trigger fibrotic responses (Greaney et al., 2023). Synthetic polymers like PCL and PLGA may initially offer rigidity but often degrade prematurely, leading to high failure rates due to foreign body reactions and excessive granulation tissue formation (Pepper et al., 2019; Liang et al., 2025). Although 3D-bioprinted hybrids improve precision, they face issues such as limited rheological consistency, cell viability, lack of functional microvascular networks, and weakening under cyclic loading, which can result in complications like tracheomalacia (Wei et al., 2024; Huang et al., 2016).

Addressing these limitations requires the development of multi-scale hybrid systems combining self-healing elastomers with ECM-based bioinks, using artificial intelligence (AI)/machine learning (ML) to optimize scaffold architecture and establishing performance benchmarks for stiffness and degradation (Guo et al., 2023; Yu et al., 2025). Advanced bioreactor-based preconditioning will be essential for creating vascularized, functional epithelium to improve graft integration (Lin et al., 2009). Progress towards GMP-compliant, patient-specific constructs that replicate native tracheal biomechanics and durability is crucial for achieving sustained clinical success (Elliott et al., 2017).

A transformative advancement in this domain is the integration of artificial intelligence (AI)/machine learning (ML) into 3D bioprinting tracheal scaffold design, offering the potential to overcome trial-and-error limitations through predictive mathematical/computational modeling and multi-objective optimization (Ramesh et al., 2024). However, major challenges remain, including the computational complexity of capturing bioink–printing–tissue interactions and the lack of validated models that correlate design parameters with long-term *in vivo* outcomes such as graft patency and epithelial regeneration. These gaps currently limit the predictive reliability, reproducibility, and clinical translatability of AI/ML-based scaffold design approaches (Kong et al., 2025). From a future-perspective viewpoint, AI and ML represent the next major step in advancing tracheal scaffold design, enabling the interpretation of medical imaging, the creation of patient-specific graft geometries, and the data-driven optimization of bioprinting parameters such as temperature and extrusion rate (Zhang et al., 2025). This transition from intuition-based experimentation to fully data-driven scaffold geometry optimization, biomaterial selection, and determination of printing parameters is significantly accelerated by AI, enabling biomaterials discovery and predictive modeling of tissue maturation. Looking ahead, AI-driven quality control systems and generative design tools will further automate and customize the production of complex tracheal constructs (Zhang et al., 2024a). ML models (e.g. random forest, neural network, etc.) trained on comprehensive age/gender-specific datasets linking scaffold architecture, bioink formulations, and fabrication conditions to biological outcomes, such as mechanical anisotropy, mucosal regeneration, and vascularization, can identify optimal design parameters even before fabrication begins (Zhang et al., 2024a; Ramesh et al., 2024). Deep learning algorithms may be used for real-time monitoring of cell distribution and tissue formation, enabling rapid design iterations that more closely mimic native anatomy (Sriganesh, 2025). Additionally, generative design and topology-optimization approaches have the potential to create novel chondro-fibrous ring configurations and porous architectures that preserve airway rigidity, enhance flexibility, and reduce stress concentrations during physiological loading. Additionally, combining AI with advanced bioprinting techniques, such as hybrid and 4D bioprinting, could lead to adaptive tracheal grafts that remodel with the host, paving the way for effective lifelong airway regeneration (Javaid et al., 2025). Hence, this AI-enhanced design framework has the potential to profoundly accelerate the development of more reliable, functional, and clinically translatable tracheal grafts.

To translate these advances from bench to bedside, factors such as scalability, regulatory compliance, and good manufacturing practices (GMP) must be addressed. Regulators must stay ahead of these innovations to effectively manage the regulatory landscape surrounding tracheal tissue-engineered patches and 3D bioprinted tracheal implants. This landscape is complex and multi-dimensional, requiring stringent measures to ensure the safe, effective, and ethical use of these products. Essential actions include rigorous testing, adherence to high manufacturing standards, continuous monitoring, and post-market surveillance. Regulatory frameworks must keep pace with the urgent need to deliver real clinical benefits for patients, especially within the broader context of regenerative medicine. A 3D-bioprinted living tracheal construct containing viable epithelial progenitors and chondrocytes faces far greater regulatory scrutiny from the FDA and EMA than acellular scaffolds. Classified as an ATMP under EU Regulation 1394/2007 or as a biologic requiring a BLA/IND in the United States, such constructs must demonstrate post-printing cell viability above 80%, validated cell potency, and genetic stability across 3D bioprinting variability.

In contrast to decellularized matrices, which may qualify as 361 HCT/Ps if minimally manipulated and require only donor screening and sterility testing under 21 CFR 1271, living grafts require comprehensive CMC documentation, including bioink rheology validation, reproducible bioreactor maturation, and proof of scalable GMP manufacturing, all complicated by batch-to-batch cell variability and shear-related apoptosis during multi-material printing (Sekar et al., 2021; Perin et al., 2025). While European Medicines Agency (EMA) hospital exemptions (Article 17) provide limited non-routine clinical access with strict traceability and annual safety reporting, the FDA’s RMAT designation accelerates review only after Phase I data from orthotopic large-animal studies demonstrate at least 6 months of patency without stenosis (Sekar et al., 2021). Whereas simple synthetic stents face the least regulatory complexity, they are typically classified as Class II medical devices under the FDA’s 510(k) pathway. They rely on substantial equivalence to predicate devices such as Dumon stents, supported by data on radial force greater than 5N, degradation (if bioresorbable, 12–24 months), and short-term patency (Alraiyes et al., 2019). As a result, they require only bench testing, limited animal irritation studies, and post-market surveillance, avoiding the extensive genotoxicity, tumorigenicity, and biodistribution testing mandated under ICH Q5D/Q11 for living constructs.

In contrast, bioprinted tracheal grafts/patches trigger dual jurisdiction CBER/CDRH due to integrated cells/devices, demanding risk-based CMC controls for printing parameters such as nozzle diameter of 200–400 μm, cell viability greater than 90%, real-time process analytical technology (PAT) for layer fidelity, and Phase I endpoints tracking immune rejection alongside functional metrics absent in stent approvals. Consequently, development timelines increase from 1 to 2 years to 8–12 years or more, and costs rise by a factor of 10–50 (Morrison et al., 2015; Mathur et al., 2025). By adopting an adaptive and proactive approach, scientists are making strides toward developing bioengineered tracheal implants for lifesaving clinical applications.
